# Supplementary figures and images for: Dynamic interplay of autophagy and membrane repair during Mycobacterium tuberculosis Infection
Source: PLoS Pathog. 2025 Jan 2;21(1):e1012830. doi: 10.1371/journal.ppat.1012830 (PMC11731705; doi:10.1371/journal.ppat.1012830)

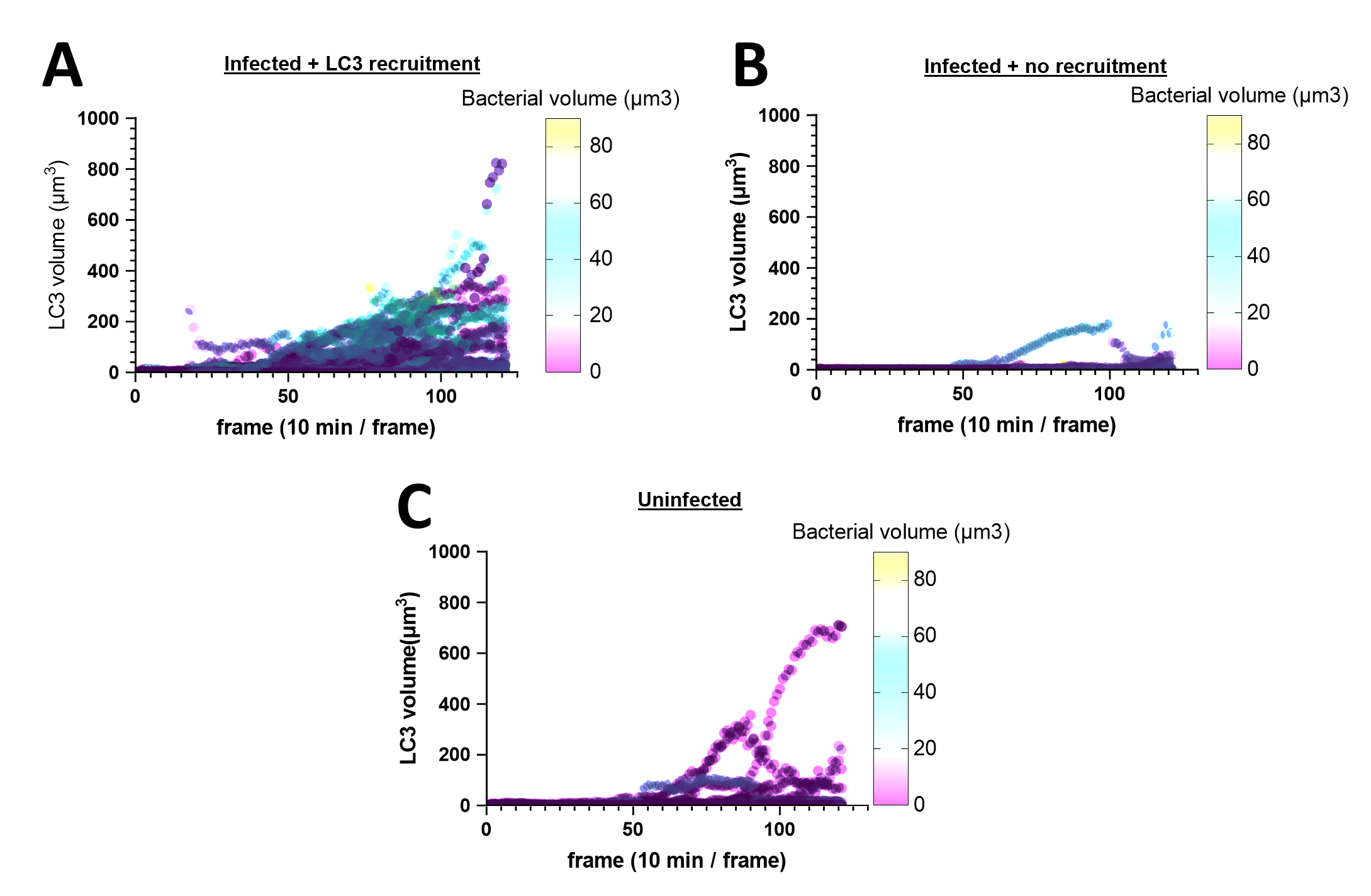

Supplement: S1 Fig — Single cell tracking and quantification of LC3 volume over time plotted on a 3 variables graph with the bacterial volume as a color dimension. Each dot represents the measurement in 1 cell in 1 frame. (A) Result of the quantification in cells exhibiting at least one temporary LC3 recruitment on at least one MCV (Ncells = 41, Npoints = 4299). (B) Quantification in cells that don’t exhibit LC3 recruitment (Ncells = 11, Npoints = 1222). (C) Quantification in uninfected cells (Ncells = 32, Npoints = 3349). (TIF) [file ppat.1012830.s001.tif]

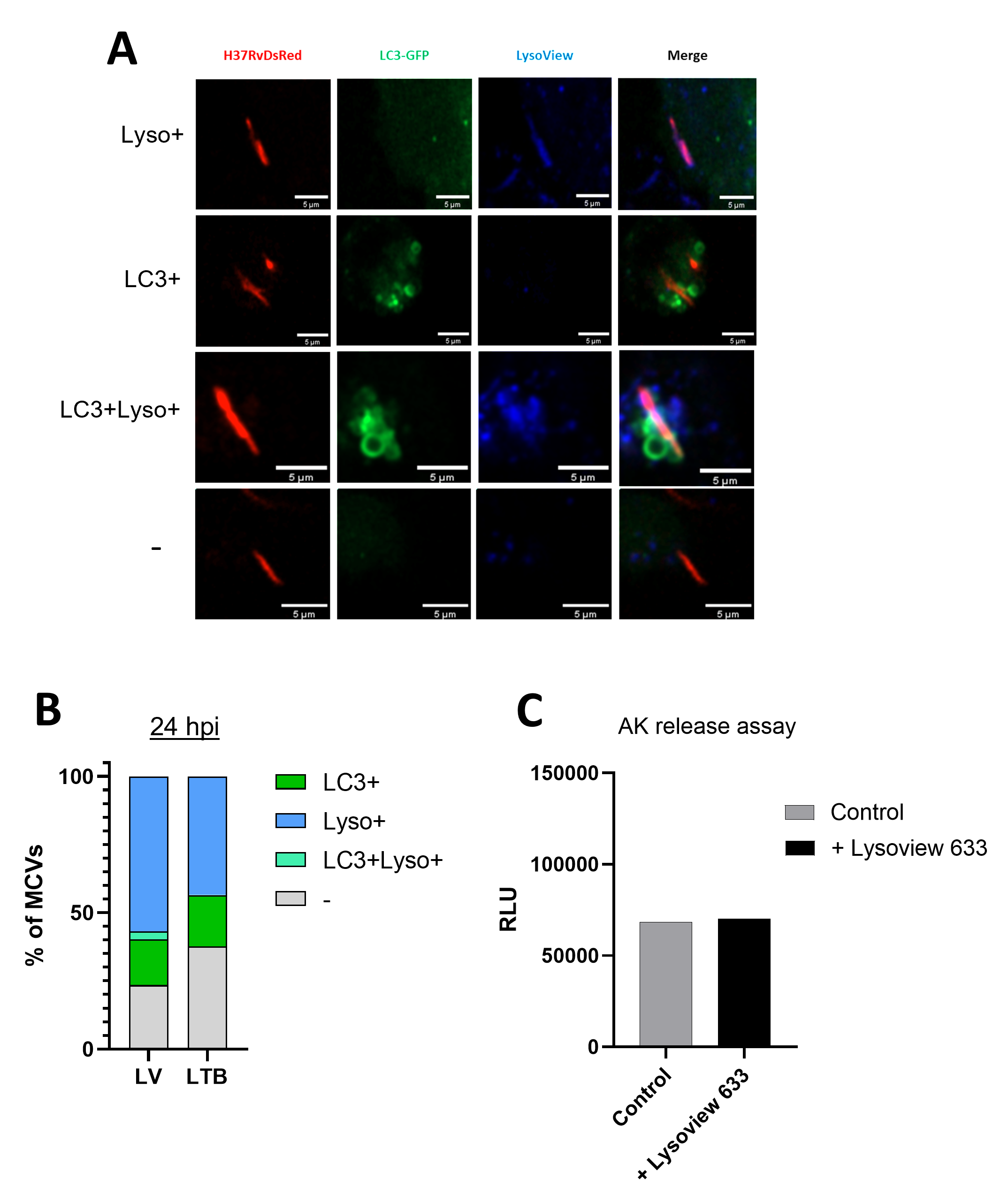

Supplement: S2 Fig — (A) Representative images of MCVs in THP-1- GFP-LC3 cells negative (-), positive for Lysoview 633 (Lyso+), for LC3 (LC3+), and double positive (LC3+Lyso+). (B) quantification of the fraction of MCVs in THP-1 cells stained for lysoview 633 or lysotracker blue. (C) The cell death level was quantified by the adenylate Kinase (AK) release assay on THP-1- GFP-LC3 treated or not with Lysoview 633 1:2000 for 48h. RLU = relative luminescence unit. (TIF) [file ppat.1012830.s002.tif]

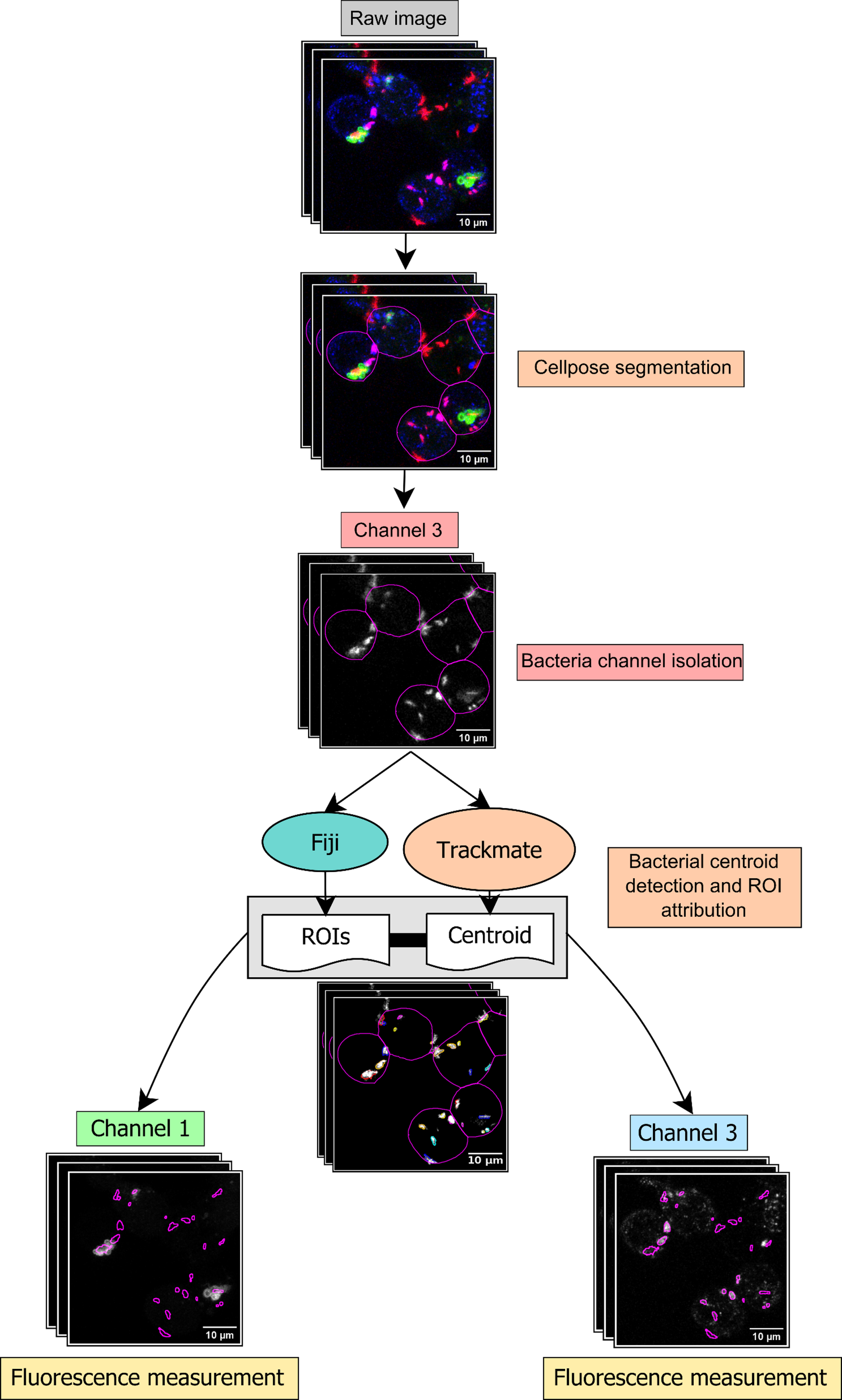

Supplement: S3 Fig — The cells are first segmented using Cellpose. The ROIs obtained are applied on the bacterial channel to retain the signal only in living cells. The bacterial signal is then segmented on Fiji to create ROIs and measure the centroids coordinates, and analyzed using Trackmate to obtain the coordinates of the MCVs’ centroids. Their distance to the ROIs centroids is calculated to retain the closest using the nearest neighbor calculation. The selected ROIs were finally applied on the channels of interest and the mean fluorescence intensity is finally measured. (TIF) [file ppat.1012830.s003.tif]

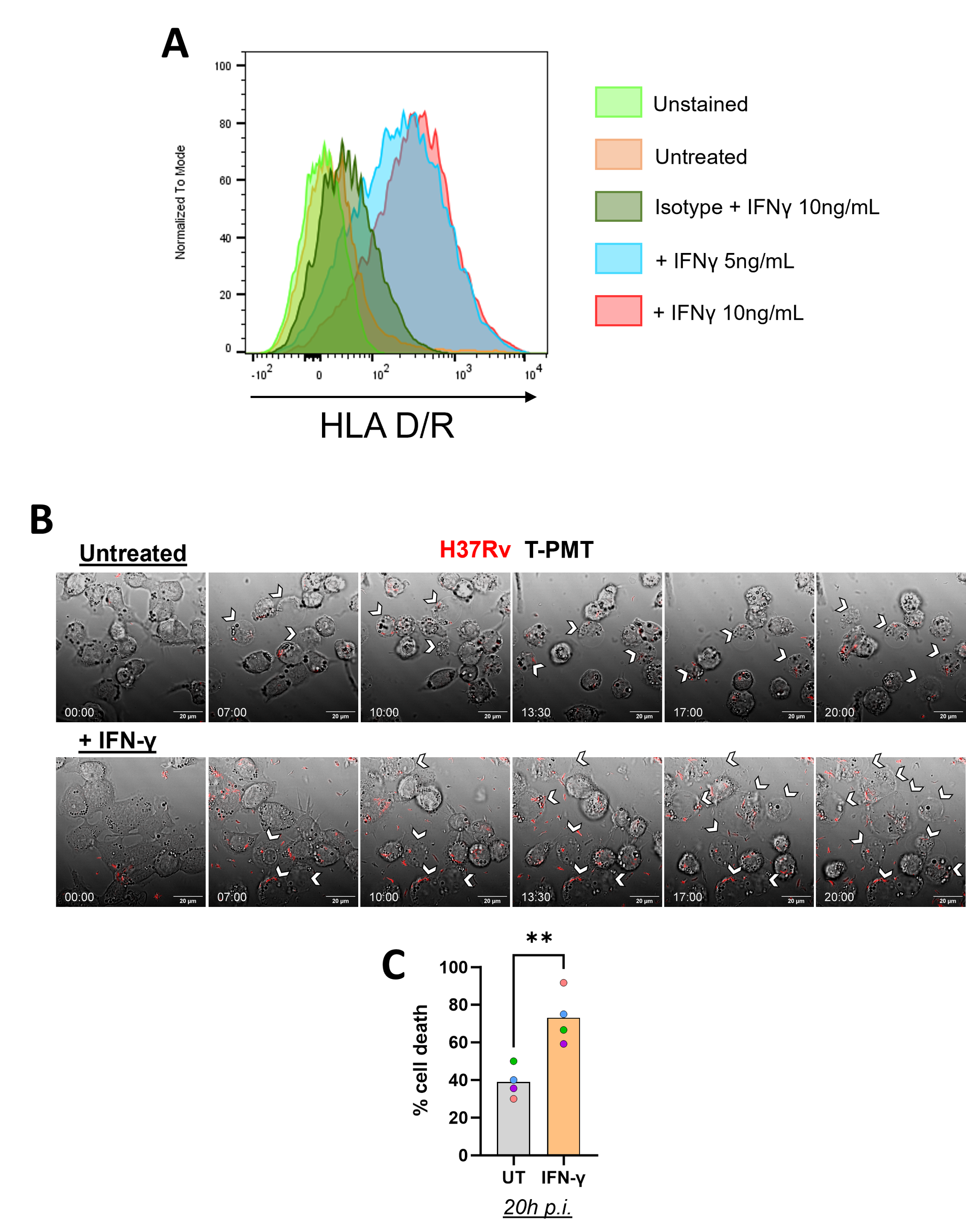

Supplement: S4 Fig — THP-1 cells were treated with IFN-γ 5 ng/mL, 10 ng/mL overnight or left untreated. Cells were fixed and stained for MHC-II expression using HLA/DR antibody (BD). Some Treated cells with 10 ng/mL were stained with isotype control. The staining was analyzed by flow cytometry. (B) Representative time lapse showing the untreated cells (top panel) or pre-stimulated with IFN-γ (bottom panel). Time stamp format is hh:mm. Chevrons are showing cells presenting the ballooning phenotype. (C) The cell death frequency was quantified by counting the proportion of cells exhibiting the ballooning phenotype during infection. Data are from 4 independent experiments. Data points color indicate the same experiment. Data between untreated and IFN-γ treated groups were compared by paired t-test. **p< 0.01. (TIF) [file ppat.1012830.s004.tif]

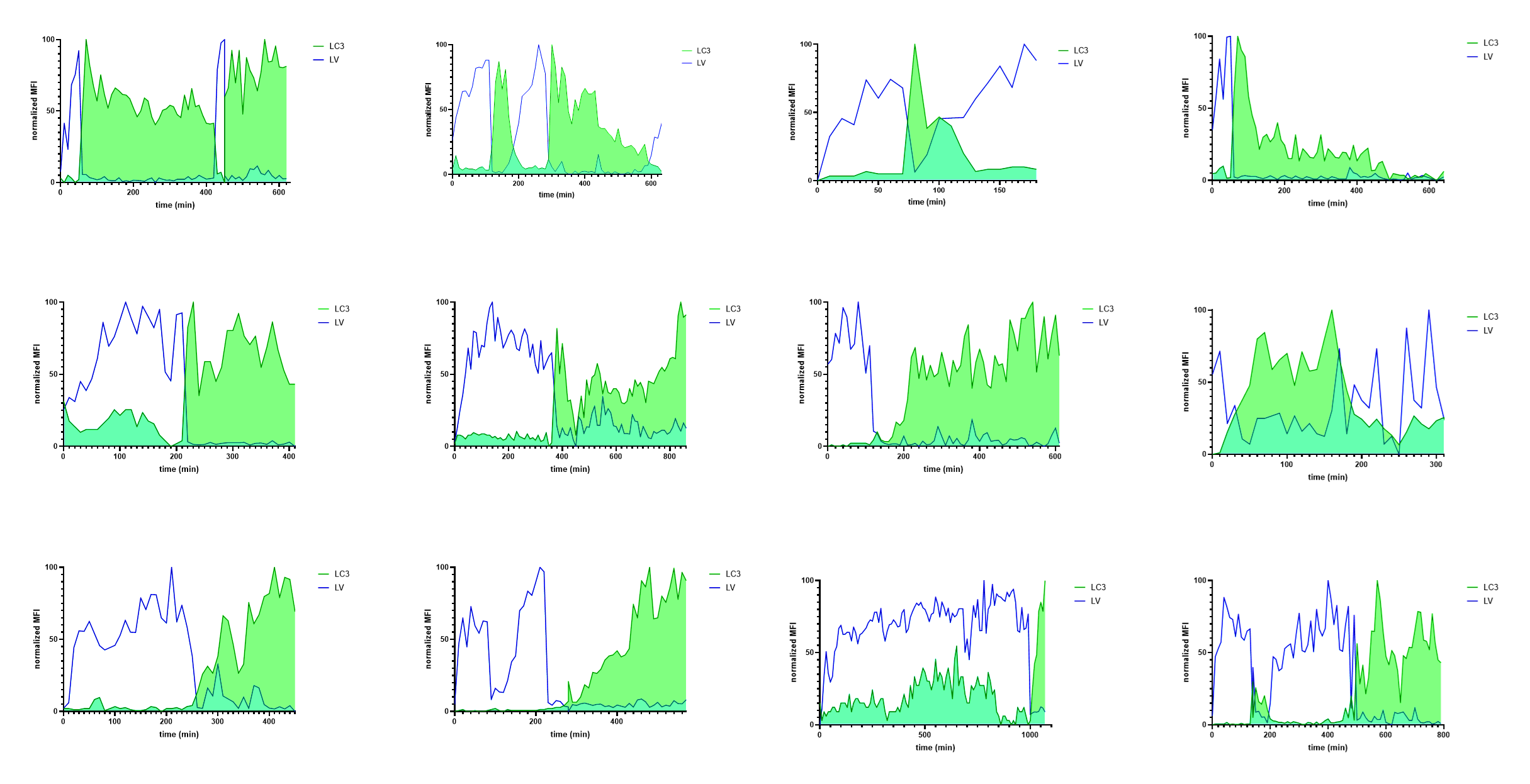

Supplement: S5 Fig — Compilation of the LC3 and lysoview fluorescence quantification on individual MCVs isolated from 4 independent experiments. Only the MCV exhibiting LC3 recruitment for more than 3 h were retained. (TIF) [file ppat.1012830.s005.tif]

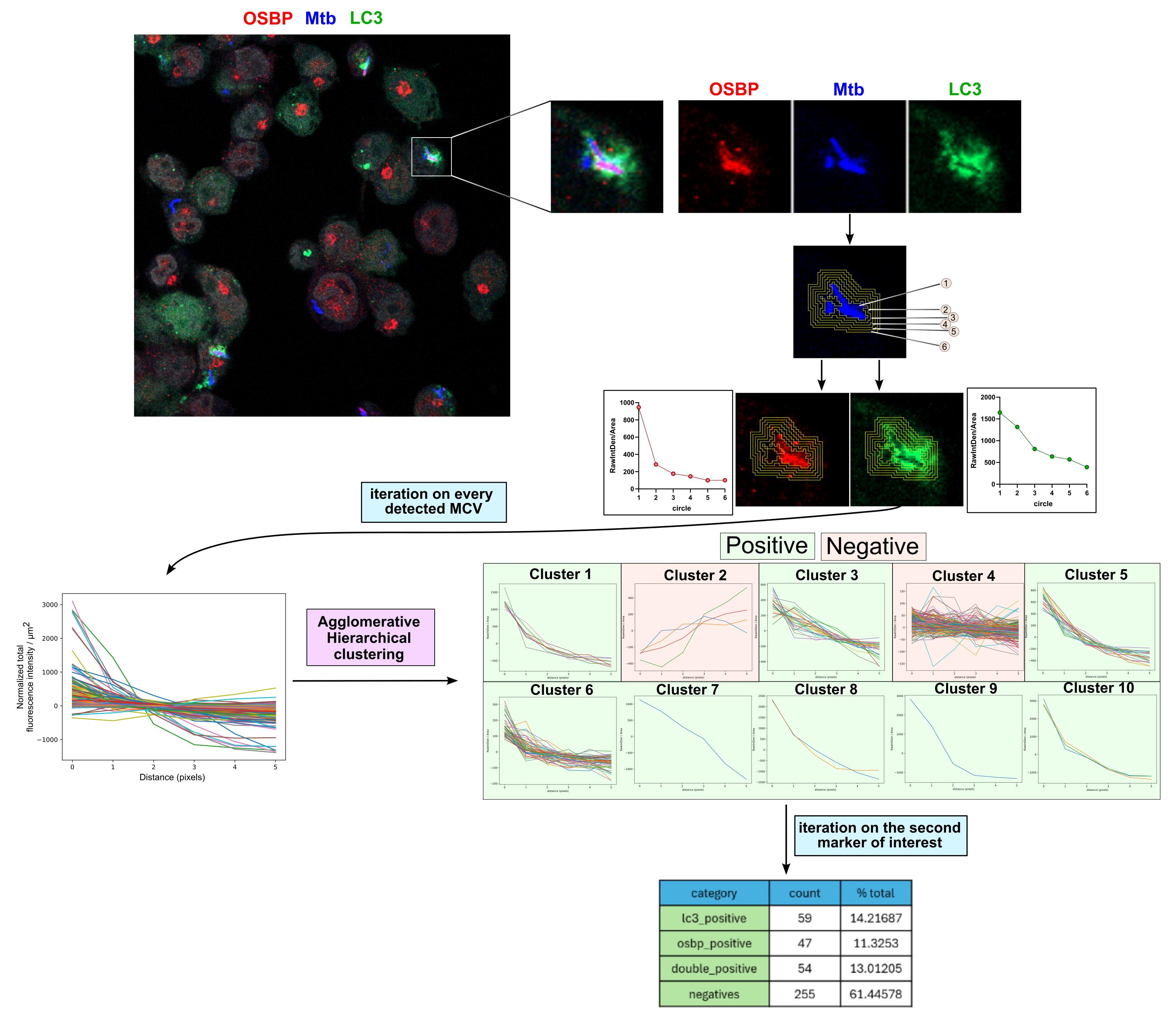

Supplement: S6 Fig — From multi-channel image, the MCVs are detected using Trackmate and Fiji as described in S1 Fig. The obtained ROI around the MCV (noted 1) is then enlarged by 1 pixel. The new ROI is enlarged by 1 pixel and the operation is repeated until 5 concentric ROIs are created around the original MCV ROI. The total fluorescence can be measured in between concentric circles to create a curve of fluorescence representing the distribution of fluorescence on and around the LCV. Each curve is then normalized by subtracting their respective average value. The pool of curves is then classified into 10 clusters using agglomerative hierarchical clustering. The cluster are then defined as positive or negative based on the shape of the cluster. Curves showing a decreasing fluorescence intensity to a plateau value were considered positive for the marker. Curves showing a flat or increasing fluorescence intensity were considered negatives for the marker. The analysis was repeated for each marker analyzed on the image. The positivity and negativity for each marker per MCV was then determined. (TIF) [file ppat.1012830.s006.tif]

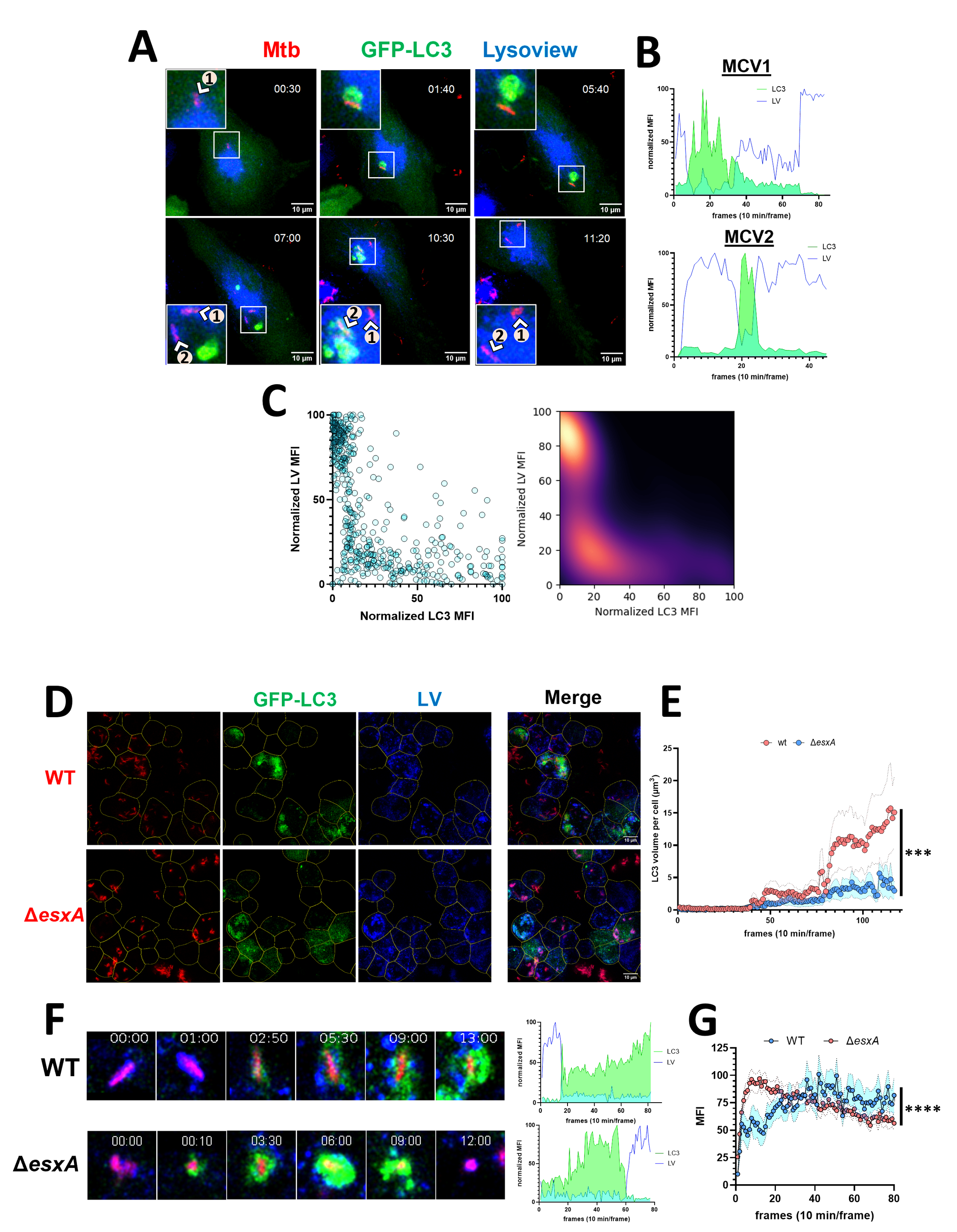

Supplement: S7 Fig — (A-C) hMDMs expressing GFP-LC3 were stained with Lysoview-633, infected with DsRed expressing Mtb at an MOI 1 and imaged by time-lapse confocal imaging for 20 h. (A) Representative image of an infected macrophage at different time point. The tracked MCV are being magnified and annotated. (B) Quantification of fluorescence at the MCV for each time point. Mean fluorescence intensity (MFI) of GFP and Lysoview (LV) was measured and normalized between values of 0 and 100 as 0 the minimum value recorded, and 100 the maximum value. (C, left panel) Quantification of LV and LC3 normalized MFI and was compared. Each dot represents the GFP and LV normalized MFI at one time point for one MCV from 2 independent experiments (n = 10 MCVs). (C, right panel) The distribution of data points in left panel is shown as a density map. (D-G) THP-1-GFP-LC3 cells were infected with Mtb WT or ΔesxA bacteria at an MOI 2 and imaged by time-lapse confocal imaging for 20 h. (D) Representative images of infected cells by Mtb WT or ΔesxA at 6 h post infection (E) Single cell tracking of infected cells and quantification of LC3 volume over time. The points represent the average LC3 volume per cells, and the ribbon the standard error to the mean (N = 2, n(cells) = 47 for WT and n(cells) = 32 for ΔesxA. (F) Representative frames of MCVs from WT (top) and ΔesxA (bottom) showing LC3 recruitment. The associated normalized MFI of GFP-LC3 and LV was plotted (right). (G) MCVs from WT and ΔesxA that were trackable for at least 3 h that did not show LC3 recruitment were followed and the LC3 MFI was quantified on the MCV. The graph displays the means and standard error to the mean (N = 2, n(WT) = 10, n(ΔesxA) = 12). The difference between the curves was analyzed by modified chi-squared method. ***p<0.01, **** p<0.001. (TIF) [file ppat.1012830.s007.tif]

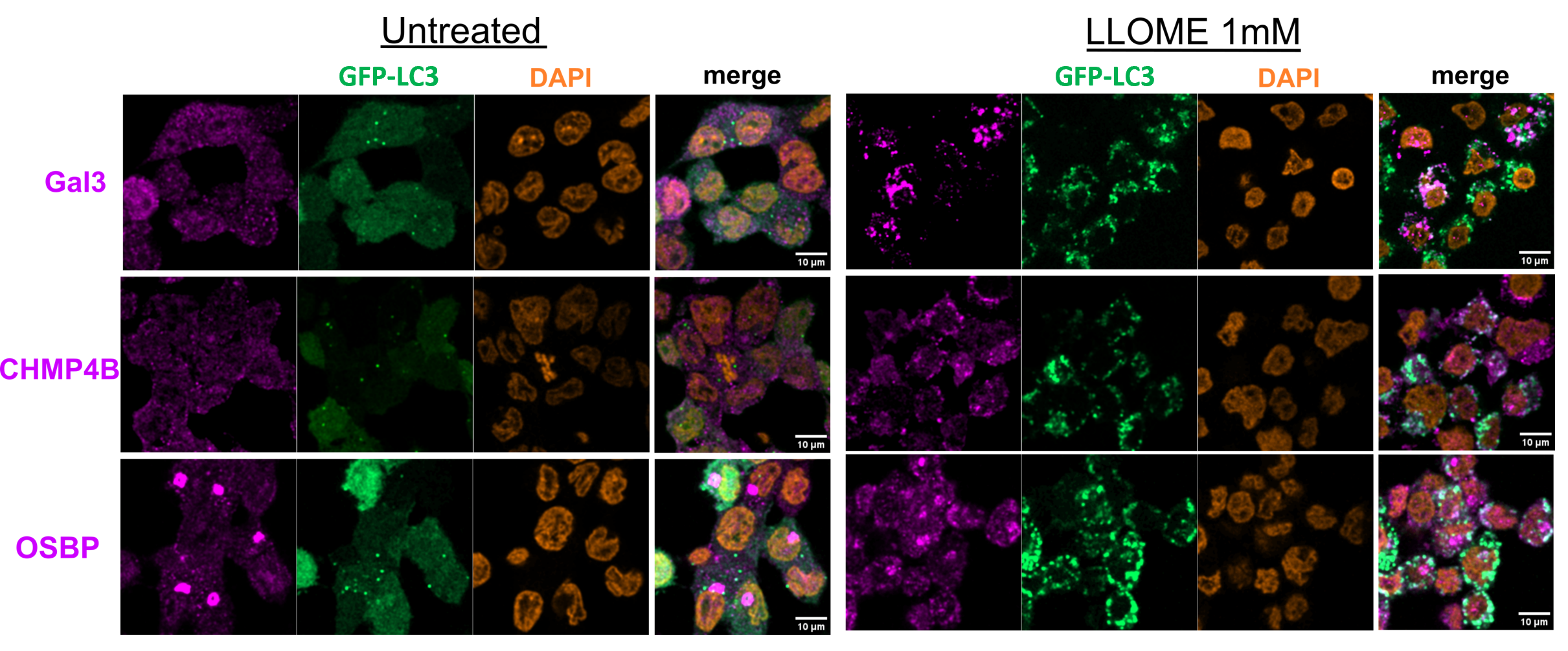

Supplement: S8 Fig — Differentiated THP-1-GFP-LC3 cells were treated with LLOMe at 1μM for 45 minutes, fixed and stained with antibodies against the indicated target. The cells were imaged using confocal microscopy. The figure displays representative images of cells untreated or treated with LLOMe. (TIF) [file ppat.1012830.s008.tif]

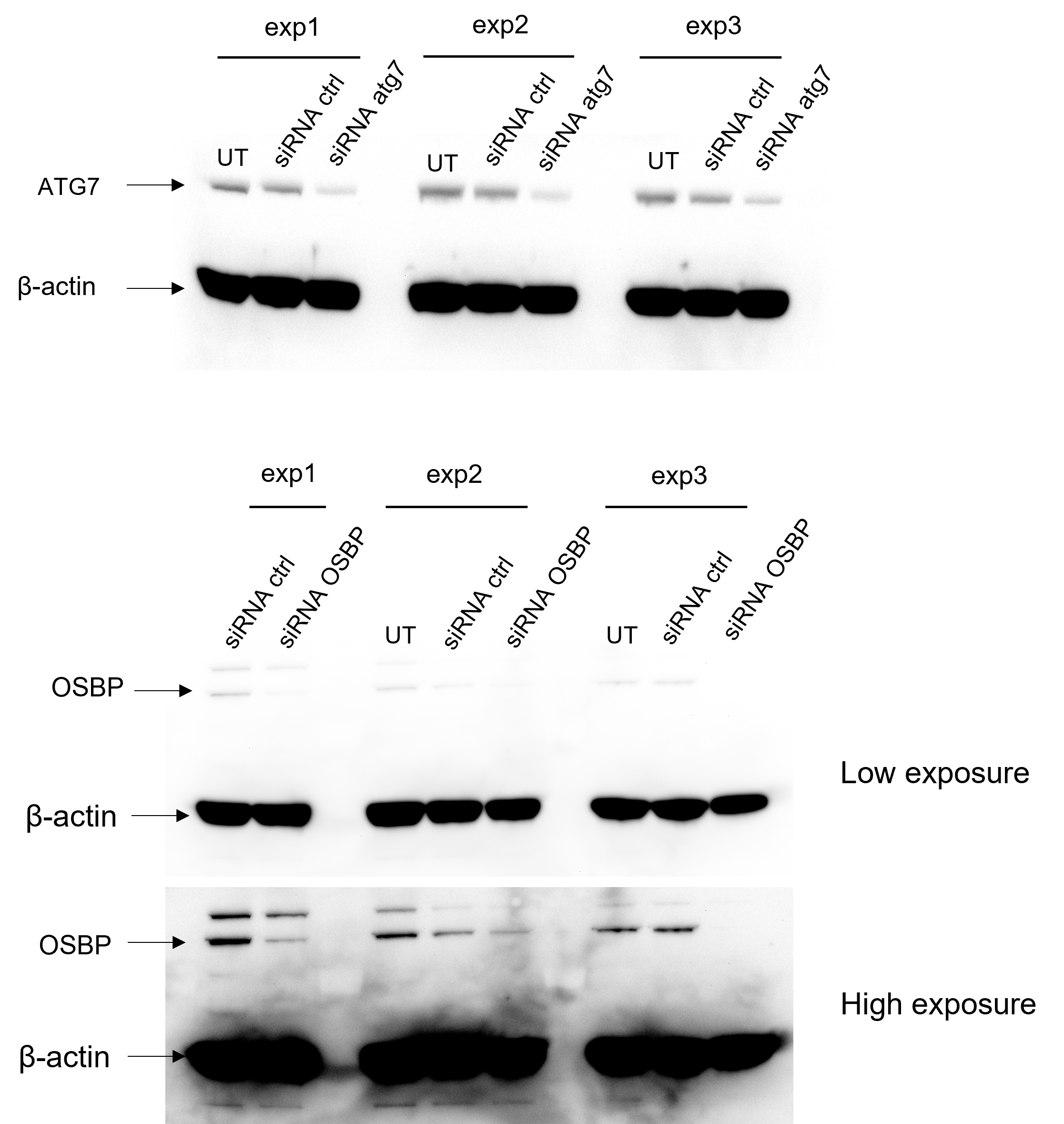

Supplement: S9 Fig — The cells were lysed, and the lysate was analyzed by SDS-PAGE followed by western blot. The β-actin blotting served as a loading control. (top) Western blotting results from cells that were left untreated, treated with negative control siRNA, and siRNA against ATG7. The lysates correspond to the cells that were used for infection in Fig 6C–6D. (Bottom) Western blotting results from cells untreated, treated with negative control siRNA, and siRNA against OSBP. The lysates correspond to the cells that were used for infection in Fig 6E–6F. A low exposure and a high exposure are shown for clarity in the relative abundance of OSBP compared to the β-actin loading control. (TIF) [file ppat.1012830.s009.tif]

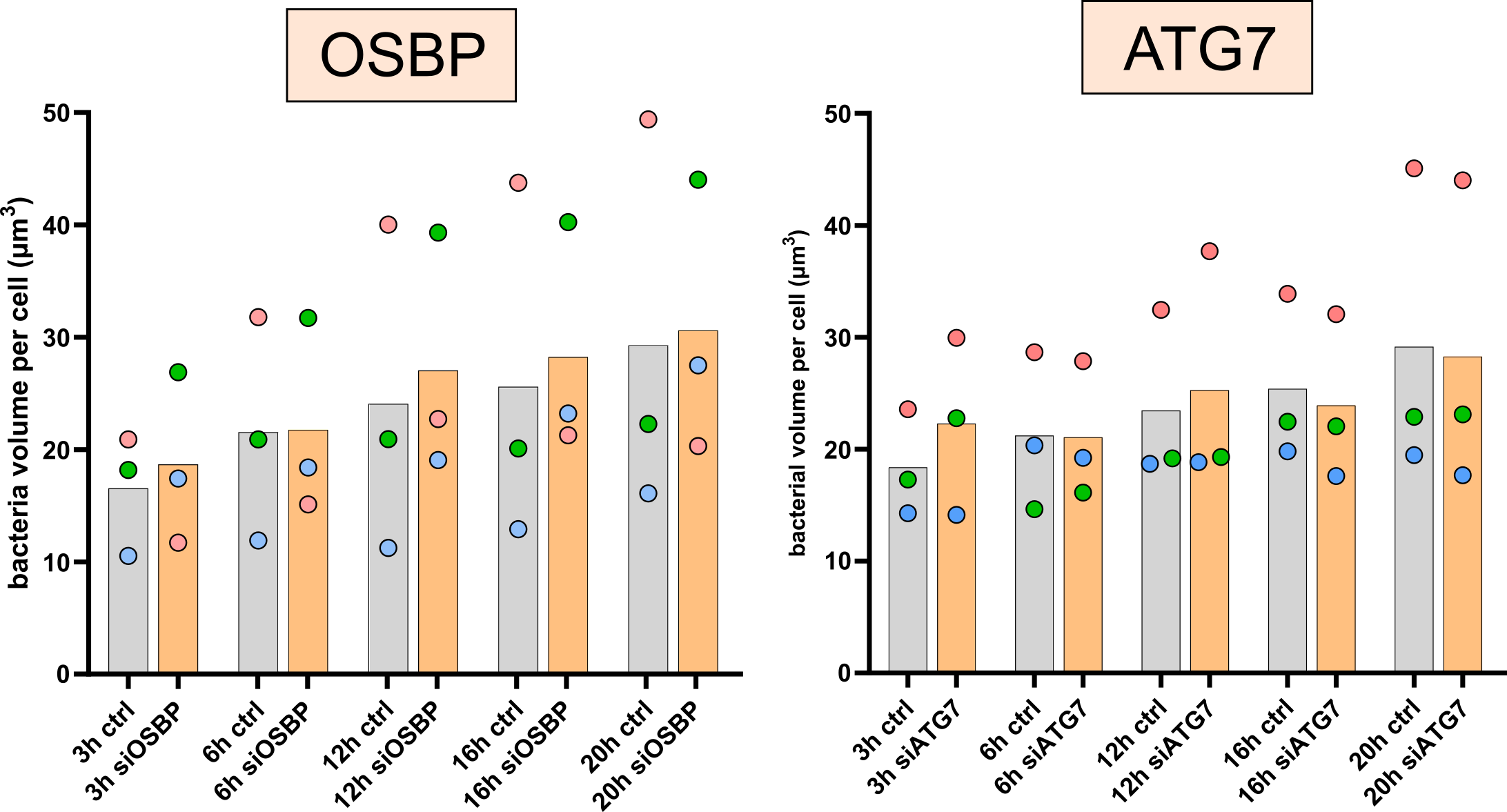

Supplement: S10 Fig — THP-1-GFP-LC3 cells were transfected with siRNA against OSBP (left) or ATG7 (right), infected with Mtb, stained by Lysoview-633 and imaged by time-lapse confocal microscopy. The relative bacterial burden was determined by calculation of the bacterial volume per cell (see methods). These results correspond to the raw values presented in the Fig 6D and 6F. The dots correspond to the average bacterial volume per cell and the color corresponds to the different independent experiments. (TIF) [file ppat.1012830.s010.tif]
