# Supplementary material for: Dynamic interplay of autophagy and membrane repair during Mycobacterium tuberculosis Infection
Source: PLoS Pathog. 2025 Jan 2;21(1):e1012830. doi: 10.1371/journal.ppat.1012830 (PMC11731705; doi:10.1371/journal.ppat.1012830)
Supplement: S2 Code — The workflow is also illustrated in S6 Fig. (PDF) [file ppat.1012830.s012.pdf]

single_bact_quant_2 (1)

November 12, 2024

0.0.1 Modules and libraries

```
[ ]: from cellpose import models, io
from cellpose.io import *
from collections import defaultdict
import geopandas
import glob
import imagej
from jpype import JArray, JInt
import matplotlib.pyplot as plt
import multiprocessing as mp
import numpy as np
import os
import re
import pandas
from pandas import DataFrame
from pathlib import Path
import scyjava
import seaborn
import shutil
import tkinter as tk
from tkinter import filedialog
from PIL import Image
import sys
from zipfile import ZipFile
import csv
import random
from math import isnan
from sklearn.cluster import AgglomerativeClustering
from PyQt5.QtWidgets import QApplication, QMainWindow, QWidget, QScrollArea,␣
↪QLabel, QGridLayout
from PyQt5.QtGui import QPixmap
from PyQt5.QtCore import Qt
from sklearn import decomposition
from sklearn.decomposition import PCA
from sklearn import discriminant_analysis
from sklearn.discriminant_analysis import LinearDiscriminantAnalysis as LDA
from sklearn.preprocessing import StandardScaler
```

0.0.2 Initialization of Fiji

```
[ ]: scyjava.config.add_option('-Xmx60g')
ij = imagej.init('/Users/MrWor/Documents/Fiji.app', mode='interactive')
ij.ui().showUI()
ij.getVersion()
```

```
[ ]: # Different plugin needed for the workflow
showPolygonRoi = scyjava.jimport('ij.gui.PolygonRoi')
Overlay = scyjava.jimport('ij.gui.Overlay')
```

```
Regions = scyjava.jimport('net.imglib2.roi.Regions')
LabelRegions = scyjava.jimport('net.imglib2.roi.labeling.LabelRegions')
ZProjector = scyjava.jimport('ij.plugin.ZProjector')()
Duplicator = scyjava.jimport('ij.plugin.Duplicator')()
ov = Overlay()
Model =  scyjava.jimport('fiji.plugin.trackmate.Model')
Settings= scyjava.jimport('fiji.plugin.trackmate.Settings')
TrackMate = scyjava.jimport('fiji.plugin.trackmate.TrackMate')
Settings= scyjava.jimport('fiji.plugin.trackmate.Settings')
TrackMate = scyjava.jimport('fiji.plugin.trackmate.TrackMate')
Logger= scyjava.jimport('fiji.plugin.trackmate.Logger')
DetectorKeys= scyjava.jimport('fiji.plugin.trackmate.detection.DetectorKeys')
ExportTracksToXML= scyjava.jimport('fiji.plugin.trackmate.action.
 ↪ExportTracksToXML')
TmXmlWriter= scyjava.jimport('fiji.plugin.trackmate.io.TmXmlWriter')
LogRecorder = scyjava.jimport('fiji.plugin.trackmate.util.LogRecorder')
SparseLAPTrackerFactory= scyjava.jimport('fiji.plugin.trackmate.tracking.
 ↪jaqaman.SparseLAPTrackerFactory')
TMUtils = scyjava.jimport('fiji.plugin.trackmate.util.TMUtils')
HyperStackDisplayer = scyjava.jimport('fiji.plugin.trackmate.visualization.
 ↪hyperstack.HyperStackDisplayer')
SelectionModel = scyjava.jimport('fiji.plugin.trackmate.SelectionModel')
CellposeDetectorFactory = scyjava.jimport('fiji.plugin.trackmate.cellpose.
 ↪CellposeDetectorFactory')
FeatureFilter = scyjava.jimport('fiji.plugin.trackmate.features.FeatureFilter')
DisplaySetting = scyjava.jimport('fiji.plugin.trackmate.gui.displaysettings.
 ↪DisplaySettings')
DisplaySettingsIO = scyjava.jimport('fiji.plugin.trackmate.gui.displaysettings.
 ↪DisplaySettingsIO')
CaptureOverlayAction = scyjava.jimport('fiji.plugin.trackmate.action.
 ↪CaptureOverlayAction')
PretrainedModel= scyjava.jimport('fiji.plugin.trackmate.cellpose.
 ↪CellposeSettings.PretrainedModel')
ThresholdDetectorFactory= scyjava.jimport('fiji.plugin.trackmate.detection.
 ↪ThresholdDetectorFactory')
TrackScheme = scyjava.jimport('fiji.plugin.trackmate.visualization.trackscheme.
 ↪TrackScheme')
TrackTableView = scyjava.jimport('fiji.plugin.trackmate.visualization.table.
 ↪TrackTableView')
AllSpotsTableView = scyjava.jimport('fiji.plugin.trackmate.visualization.table.
 ↪AllSpotsTableView')

rm = ij.RoiManager.getRoiManager()
```

0.0.3 Function used for the workflow

[ ]:
```python
def get_bacteria_area(bact_path, directory_path, threshold_low, threshold_high, distance, known, unit):
    # Iterate over the folders and process each folder
    w = scyjava.jimport('ij.WindowManager')
    file_pattern = os.path.join(directory_path, "*.czi")
    file_list = glob.glob(file_pattern)
    for file_path in file_list:
        rm.runCommand("Reset")
        basename = os.path.basename(file_path)
        corename = os.path.splitext(basename)[0]
        image_path = f"{bact_path}bact_channel_{corename}.tif"
        basename2 = os.path.basename(image_path)
        imp = ij.IJ.openImage(image_path)
        print(f"Opening {image_path}")
        ij.IJ.run(imp, "Smooth", "stack")
        ij.IJ.run(imp, "Smooth", "stack")
        imp.show()
        ij.py.run_macro(f'run("Set Scale...", "distance={distance} known={known} unit={unit}");')
        ij.IJ.run("Set Measurements...", f'area limit redirect=None decimal=2')
        ij.IJ.setAutoThreshold(imp, "Default dark")
        ij.IJ.setRawThreshold(imp, threshold_low, threshold_high)
        ij.IJ.run(imp, "Convert to Mask", "background=Dark black");
        input_ROI = Path(f"{voronoi_path}/{corename}_voronoi.zip")
        rm.open(f"{input_ROI}")
        roi_count = rm.getCount()
        for i in range(0, roi_count-1):
            rm.select(i)
            ij.IJ.run(imp, "Analyze Particles...", f"summarize stack");
        window_name = f"Summary of {basename2}"
        ij.IJ.selectWindow(window_name)
        ij.IJ.renameResults(f"{window_name}", "Results")
        output_path = Path(f"{bact_path}{corename}_area_summary.csv").as_posix()
        ij.IJ.saveAs("Results", output_path)
        ij.IJ.run("Clear Results")
        w.getWindow("Results").close()
        rm.runCommand("Reset")
        ij.py.run_macro('close("*");')


def track_bacteria(directory_path, bact_path, dsettings, quality_filter, tsettings, display=False):
    '''
    This function leverages ImageJ's Trackmate plugin to track the bacteria within each single-cell video provided in the input folder bact.
```

```
    It uses the Tresholding Detector for segmentation (with detection␣
↪parameters specified in dsettings), and the LAP Tracker algorithm for␣
↪tracking (with tracking parameters specified in tsettings). The function␣
↪also takes as input the desired quality filter on the detected spots.
    The function saves the Trackmate XML model as well as a CSV file of the␣
↪tracks for each cell in bact/Output/.
    '''
    out = bact_path+"Output/"
    if not os.path.exists(out):
        os.makedirs(out)
    for image in os.listdir(bact_path):
        basename = os.path.basename(image)
        if basename.startswith('bact'):
            if not image.endswith("overlay.tif"):
                if (image[len(image)-4:] == ".tif"):
                    # Open Image
                    imp = ij.IJ.openImage(bact_path + image)
                    print(f"Trackmate is opening {image}")
                    imp.show()
                    ij.IJ.run(imp, "Select None", "")
                    ij.py.run_macro(f'run("Set Scale...", "distance={distance}␣
↪known={known} unit={unit}");')
                    ij.IJ.run("Smooth", "stack")
                    ij.IJ.run("Smooth", "stack")
                    # Create Model
                    model = Model()
                    settings = Settings(imp)
                    # Detector
                    settings.detectorFactory = ThresholdDetectorFactory()
                    for parameter, value in dsettings.items():
                        settings.detectorSettings[parameter] = value
                    filter1 = FeatureFilter('QUALITY', quality_filter, True)
                    settings.addSpotFilter(filter1)
                    #print(settings.detectorSettings)
                    # Tracker
                    settings.trackerFactory = SparseLAPTrackerFactory()
                    settings.trackerSettings = settings.trackerFactory.
↪getDefaultSettings()
                    for parameter, value in tsettings.items():
                        settings.trackerSettings[parameter] = value
                    # Execute Tracking
                    trackmate = TrackMate(model, settings)
                    ok = trackmate.checkInput()
                    if not ok:
                        sys.exit(str(trackmate.getErrorMessage()))
                    ok = trackmate.process()
                    if not ok:
```

```
                        #sys.exit(str(trackmate.getErrorMessage()))
                        print(str(trackmate.getErrorMessage()))
                        ij.py.run_macro('close("*");')

                    selectionModel = SelectionModel(model)
                    # Display
                    ds = DisplaySettingsIO.readUserDefault()
                    if display:
                        displayer = HyperStackDisplayer(model, selectionModel,␣
↪imp, ds)

                        displayer.render()
                        displayer.refresh()
                        trackscheme = TrackScheme(model, selectionModel, ds)
                        trackscheme.render()
                    # Save Data
                    basename_image = os.path.basename(image)
                    corename = os.path.splitext(basename_image)[0]
                    outFile = Path(out+corename+"_exportModel.xml")
                    writer = TmXmlWriter(outFile)
                    writer.appendModel(model)
                    writer.appendSettings(settings)
                    writer.writeToFile()
                    csvFileSpots = out+corename+"_exportTracks.csv"
                    spotsTableView = AllSpotsTableView(model, selectionModel,␣
↪ds, image)
                    spotsTableView.exportToCsv(csvFileSpots)
                    ij.py.run_macro('close("*");')

def merge_trackmate_rois(bact_path, directory_path, grouped_SC):
    '''
    This function seeks to assign bacterial ROIs to tracks by merging the␣
↪results of tracking (via Trackmate) and ROI detection (via Thresholding in␣
↪ImageJ). It matches ROIs to spots detected in Trackmate based on the nearest␣
↪centroid.
    For each cell, the function saves a CSV file of tracks and their␣
↪corresponding ROIs in a folder called grouped.
    '''
    file_pattern = os.path.join(bact_path, "*.tif")
    file_list = glob.glob(file_pattern)
    for file_path in file_list:
        basename = os.path.basename(file_path)
        if basename.startswith('bact'):
            if not file_path.endswith("overlay.tif"):
                filename = os.path.basename(file_path)
                filename2 = os.path.splitext(filename)[0]
                first = Path(f"{bact_path}/Output/{filename2}_exportTracks.
↪csv").as_posix()
```

```
                second = Path(f"{bact_path}/{filename2}_bactROI.csv").as_posix()
                if os.path.exists(first) and os.path.exists(second):
                    df_first = pandas.read_csv(first)
                    df_first = df_first.drop([0,1,2])
                    if len(df_first)>0:
                        print(f"{filename2}_exportTracks.csv is not empty")
                        pairwise = xref_locations(first, second,
                                                  first_x='POSITION_X',
                                                  first_y='POSITION_Y',
                                                  first_z='POSITION_Z',
                                                  second_x='X',
                                                  second_y='Y',
                                                  second_z='Slice',
                                                  max_dist=100,
                                                  verbose=False)
                        grouped = pairwise[['ID', pairwise.columns[12]]].
 ↪reset_index(drop=True)
                        grouped.rename(columns={'index_right':␣
 ↪'object_ID_list'}, inplace=True)
                        final_csv = Path(f"{grouped_SC}{filename2}_grouped.csv")
                        grouped.to_csv(final_csv)
                        print(f"Saving {final_csv}")
                        final_csv2 = Path(f"{grouped_SC}{filename2}_pairwise.
 ↪csv")
                        pairwise.to_csv(final_csv2)
                    else:
                        print(f"{filename2}_exportTracks.csv is empty")


def xref_locations(first, second, first_x='POSITION_X', first_y='POSITION_Y',␣
 ↪first_z='POSITION_Z',
                   second_x='X', second_y='Y', second_z='Slice',
                   max_dist=5, verbose=False):
    pairwise_elements = pandas.DataFrame()
    first_measurements = pandas.read_csv(first)
    first_measurements = first_measurements.drop([0,1,2])
    second_measurements = pandas.read_csv(second)
    first_gdf = geopandas.GeoDataFrame(
        first_measurements,
        geometry=geopandas.points_from_xy(first_measurements[first_x],
                                          first_measurements[first_y],
                                          first_measurements[first_z]))
    second_gdf = geopandas.GeoDataFrame(
        second_measurements,
        geometry=geopandas.points_from_xy(second_measurements[second_x],
                                          second_measurements[second_y],
                                          second_measurements[second_z]))
```

```python
    ti_rows = first_gdf.shape[0]
    tj_rows = second_gdf.shape[0]
    for ti_row in range(0, ti_rows):
        if verbose:
            print(f"On row: {ti_row}")
        ti_element = first_gdf.iloc[[ti_row, ]]

        titj = geopandas.sjoin_nearest(ti_element, second_gdf,
                                       distance_col="pairwise_dist",
                                       max_distance=max_dist)
        chosen_closest_dist = titj.pairwise_dist.min()
        if (isnan(chosen_closest_dist)):
            print(f"This element has no neighbor within {max_dist}.")
        else:
            chosen_closest_cell = titj.pairwise_dist == chosen_closest_dist
            chosen_closest_row = titj[chosen_closest_cell]
            pairwise_tmp = pandas.concat([pairwise_elements, chosen_closest_row])
            pairwise_elements = pairwise_tmp
    return pairwise_elements

def apply_bact_overlays(bact_path, grouped):
    '''
    This function allows for visual quality-control of the bacterial-tracking step by adding colored overlays to the bacterial tracks in each single-cell video. Bacterial clusters of the same track remain the same color from frame to frame.
    All of the overlayed videos are saved in the folder bact with the title [cell_number]_overlay.tif.
    '''
    file_pattern = os.path.join(bact_path, "*.tif")
    file_list = glob.glob(file_pattern)
    colors = ["blue", "cyan", "green", "magenta", "orange", "red", "yellow"]
    for file_path in file_list:
        f_name = os.path.basename(file_path)
        if f_name.startswith('bact'):
            if not file_path.endswith("overlay.tif"):
                f_name = os.path.basename(file_path)
                print(f"opening {f_name}")
                f_name = os.path.splitext(f_name)[0]
                imp = ij.IJ.openImage(file_path)
                ij.ui().show(imp)
                ij.IJ.run(imp, "Select None", "")
                input_csv = Path(f"{grouped}/{f_name}_grouped.csv")
                if os.path.exists(input_csv):
                    df = pandas.read_csv(input_csv)
                    pouet = df['object_ID_list']
```

```
                    input_ROI = Path(f"{bact_path}/{f_name}_bactROI.zip")
                    if not os.path.exists(input_ROI):
                        input_ROI = Path(f"{bact_path}/{f_name}_bactROI.roi")
                    rm.open(f"{input_ROI}")
                    for i in range(len(pouet)):
                        cell = df['object_ID_list'][i]
                        cell_index = int(cell)
                        random_color = random.choice(colors)
                        rm.select(cell_index)
                        print(f"selecting ROI {cell_index}")
                        imp.getRoi()
                        roi = imp.getRoi()
                        if roi is None:
                            roi_number = ij.py.to_java(str(cell_index))
                            selection = f"roiManager('Select', {roi_number});"
                            ij.py.run_macro(selection)
                        overlay_command = f"Overlay.
↪addSelection('{random_color}',2);"
                        ij.py.run_macro(overlay_command)
                    ij.py.run_macro("setMinAndMax(0, 100);")
                    ij.py.run_macro("run('Flatten', 'stack');")
                    method = 'max all'
                    z_projector_result = ZProjector.run(imp, method)
                    z_collapsed_image = ij.py.from_java(z_projector_result)
                    z_collapsed_dataset = ij.py.to_dataset(z_projector_result)
                    result_path = os.path.splitext(file_path)[0] + "_overlay.
↪tif"
                    if os.path.exists(result_path):
                        os.remove(result_path)
                    saved = ij.io().save(z_collapsed_dataset, result_path)
                    ij.py.run_macro('close("*");')
                    rm.runCommand("Reset")
                else:
                    ij.py.run_macro('close("*");')
                    rm.runCommand("Reset")



def calculate_total_volume_per_cell(df):
    """Calculates the sum of the "Total Area" for each pattern of the "Slice"␣
↪column.

    Args:
        df: A pandas DataFrame containing the data.

    Returns:
```

```python
        A pandas DataFrame containing the sum of the "Total Area" for each␣
↪pattern of the "Slice" column.
    """

    # Create a list to store the DataFrames with results.
    results = []

    # Loop through the DataFrame.
    for i in range(len(df)):
        # If the current cell value in the "Slice" column is 1,
        if df['Slice'][i] == 1:
            # Sum the "Total Area" column for the next 10 rows.
            #slice_number = 10  # You need to define slice_number and␣
↪slice_distance
            #slice_distance = 1  # You need to define slice_number and␣
↪slice_distance
            bacterial_area = df['Total Area'][i:i + slice_number].sum()
            bacterial_volume = bacterial_area * slice_distance
            # Create a DataFrame with the current pattern number and the total␣
↪area.
            result_df = pandas.DataFrame({'bacterial_volume':␣
↪[bacterial_volume]}, index=[i])
            results.append(result_df)

    # Concatenate the list of DataFrames to create the final results DataFrame.
    final_results = pandas.concat(results, ignore_index=True)

    # Return the final results DataFrame.
    return final_results

def single_cell_isolation(cois, directory_path, bact_path, voronoi_path,␣
↪channels_path, size_limit = 1):
    file_pattern = os.path.join(directory_path, "*.czi")
    file_list = glob.glob(file_pattern)
    final_results_path = f"{directory_path}final_results/"
    for file_path in file_list:
        f_name = os.path.basename(file_path)
        corename = os.path.splitext(f_name)[0]
        single_cell_path = f"{directory_path}single_cells/{corename}/"
        if not os.path.exists(single_cell_path):
            os.makedirs(single_cell_path)
        roi_path = f"{voronoi_path}{corename}_voronoi.zip"
        burden_csv = f"{final_results_path}/{corename}_burden.csv"
        df = pandas.read_csv(burden_csv)
        rm.open(roi_path)
        for channel in cois:
```

```
            if channel.startswith("bact"):
                image_path = f"{bact_path}{channel}_{corename}.tif"
            else:
                image_path = f"{channels_path}{channel}_{corename}.tif"
            imp = ij.IJ.openImage(image_path)
            imp.show()
            #imp.setSlice(4)
            #roi_count = rm.getCount()
            #for n in range(roi_count-1):
            for index, row in df.iterrows():
                if row["bacterial_volume"] > size_limit:
                    rm.select(index)
                    cell_name = f"{channel}_{corename}_{index}"
                    ij.IJ.run("Duplicate...", f"title={str(cell_name)} duplicate")
                    ij.IJ.run("Clear Outside", "stack")
                    new_vid =  w.getImage(str(cell_name))
                    ij.IJ.save(new_vid, single_cell_path + cell_name + ".tif")
                    w.getImage(str(cell_name)).close()
            ij.py.run_macro('close("*");')
        rm.runCommand("Reset")

def single_composite_isolation(cois, directory_path, voronoi_path, size_limit = 1):
    file_pattern = os.path.join(directory_path, "*.czi")
    file_list = glob.glob(file_pattern)
    final_results_path = f"{directory_path}final_results/"
    for file_path in file_list:
        f_name = os.path.basename(file_path)
        corename = os.path.splitext(f_name)[0]
        roi_path = f"{voronoi_path}{corename}_voronoi.zip"
        burden_csv = f"{final_results_path}/{corename}_burden.csv"
        df = pandas.read_csv(burden_csv)
        rm.open(roi_path)
        imp = ij.IJ.openImage(file_path)
        imp.show()
        imp.setDisplayMode(1)
        for index, row in df.iterrows():
            if row["bacterial_volume"] > size_limit:
                cell_name = f"{corename}_{index}"
                output_path = f"{directory_path}single_cells/{corename}/rois_bact/{cell_name}/"
                if os.path.exists(output_path):
                    rm.select(index)
                    ij.IJ.run("Duplicate...", f"title={str(cell_name)} duplicate")
                    ij.IJ.run("Clear Outside", "stack")
```

```
                    new_vid =  w.getImage(str(cell_name))
                    ij.IJ.save(new_vid, output_path + cell_name + ".tif")
                    w.getImage(str(cell_name)).close()
        ij.py.run_macro('close("*");')
        rm.runCommand("Reset")

def get_bact_rois(bact_path):
    file_pattern = os.path.join(bact_path, "*.tif")
    file_list = glob.glob(file_pattern)
    for file_path in file_list:
        f_name = os.path.basename(file_path)
        if f_name.startswith('bact'):
            if not file_path.endswith('overlay.tif'):
                imp = ij.IJ.openImage(file_path)
                f_name = os.path.basename(file_path)
                f_name = os.path.splitext(f_name)[0]
                imp.show()
                ij.IJ.run(imp, "Select None", "")
                ij.IJ.run("Smooth", "stack")
                ij.IJ.run("Smooth", "stack")
                ij.py.run_macro(f'run("Set Scale...", "distance={distance}␣
↪known={known_scale} unit={unit}");')
                ij.IJ.run("Set Measurements...", f'area centroid stack␣
↪decimal=2')
                ij.IJ.setAutoThreshold(imp, "Default dark")
                ij.IJ.setRawThreshold(imp, threshold_low, threshold_high)
                ij.IJ.run(imp, "Convert to Mask", "background=Dark black");
                ij.IJ.run(imp, "Analyze Particles...",␣
↪f"size={threshold_size}-Infinity display add stack");
                #ij.IJ.run(imp, "Measure", "");
                roi_count = rm.getCount()
                if roi_count == 0:
                    print(f"this {f_name} has no bacteria")
                    ij.py.run_macro('close("*");')
                elif roi_count == 1:
                    rm.save(f"{bact_path}" + f"{f_name}_bactROI.roi")
                    output_path = Path(f"{bact_path}{f_name}_bactROI.csv").
↪as_posix()
                    ij.IJ.saveAs("Results", output_path)
                    ij.IJ.run("Clear Results")
                    ij.py.run_macro('close("*");')
                    rm.runCommand("Reset")
                else:
                    rm.save(f"{bact_path}" + f"{f_name}_bactROI.zip")
                    output_path = Path(f"{bact_path}{f_name}_bactROI.csv").
↪as_posix()
                    ij.IJ.saveAs("Results", output_path)
```

```
                    ij.IJ.run("Clear Results")
                    ij.py.run_macro('close("*");')
                    rm.runCommand("Reset")

def circlesofhell(bact_roi_path, channel, distance, known_scale, unit, imp):
    roi_pattern = os.path.join(bact_roi_path, "*.roi")
    roi_list = glob.glob(roi_pattern)
    for roi in roi_list:
        roi_basename = os.path.basename(roi)
        roi_corename = os.path.splitext(roi_basename)[0]
        open_roi = rm.open(roi)
        rm.select(0)
        enlarged = ij.IJ.run("Enlarge...", "enlarge=1 pixel")
        rm.addRoi(enlarged)
        for n in range(0,3):
            rm.select(1+n)
            enlarged = ij.IJ.run("Enlarge...", "enlarge=1 pixel")
            rm.addRoi(enlarged)
        rm.select(0)
        ij.py.run_macro(f'run("Set Scale...", "distance={distance} known={known_scale} unit={unit}");')
        ij.IJ.run("Measure")
        imp.removeScale()
        ij.IJ.run("Make Band...", "band=1")
        ij.py.run_macro(f'run("Set Scale...", "distance={distance} known={known_scale} unit={unit}");')
        ij.IJ.run("Measure")
        imp.removeScale()
        for n in range(0,4):
            rm.select(1+n)
            ij.IJ.run("Make Band...", "band=1")
            ij.py.run_macro(f'run("Set Scale...", "distance={distance} known={known_scale} unit={unit}");')
            ij.IJ.run("Measure")
            imp.removeScale()
        rm.runCommand("Reset")
        output_path = f"{bact_roi_path}/{roi_corename}_{channel}.csv"
        saving = ij.IJ.saveAs("Results", output_path)
        ij.IJ.run("Clear Results")

def saving_rois(bact_path):
    #Saving ROIs indidually into a separate folder
    file_pattern = os.path.join(bact_path, "*.tif")
    file_list = glob.glob(file_pattern)
    for file_path in file_list:
        if not file_path.endswith('overlay.tif'):
            basename = os.path.basename(file_path)
```

```
            corename = os.path.splitext(basename)[0]
            if basename.startswith("bact"):
                corename2 = corename.split("bact_channel_")[1]
                imp = ij.IJ.openImage(file_path)
                ij.ui().show(imp)
                input_csv = Path(f"{grouped_SC}/{corename}_grouped.csv")
                if os.path.exists(input_csv):
                    bact_roi_path = bact_path + f"rois_bact/{corename2}"
                    if not os.path.exists(bact_roi_path):
                        os.makedirs(bact_roi_path)
                    df = pandas.read_csv(input_csv)
                    pouet = df['object_ID_list']
                    input_ROI = Path(f"{bact_path}/{corename}_bactROI.zip")
                    if not os.path.exists(input_ROI):
                        input_ROI = Path(f"{bact_path}/{corename}_bactROI.roi")
                    rm.open(f"{input_ROI}")
                    for i in range(len(pouet)):
                        cell = df['object_ID_list'][i]
                        cell_index = int(cell)
                        rm.select(cell_index)
                        output = f"{bact_roi_path}/{cell_index}.roi"
                        saved = rm.runCommand("Save", output)
                    rm.runCommand("Reset")
                    ij.py.run_macro('close("*");')

def single_cell_fluorescence(cois, directory_path, voronoi_path, channels_path, distance, known_scale, unit, size_limit = 1):
    file_pattern = os.path.join(directory_path, "*.czi")
    file_list = glob.glob(file_pattern)
    final_results_path = f"{directory_path}final_results/"
    set_string = f'Set Measurements...'
    measure_string = f'mean area limit integrated redirect=None decimal=2'
    for file_path in file_list:
        f_name = os.path.basename(file_path)
        corename = os.path.splitext(f_name)[0]
        roi_path = f"{voronoi_path}{corename}_voronoi.zip"
        burden_csv = f"{final_results_path}/{corename}_burden.csv"
        df = pandas.read_csv(burden_csv)
        rm.open(roi_path)
        for channel_name in cois:
            image_path = f"{channels_path}{channel_name}_{corename}.tif"
            basename2 = os.path.basename(image_path)
            corename2 = os.path.splitext(basename2)[0]
            cell_list = []
            imp = ij.IJ.openImage(image_path)
            imp.show()
            imp.setSlice(4)
```

```
            duplicated = ij.IJ.run("Duplicate...", "slice")
            duplicated_image = f"{corename2}-1.tif"
            ij.IJ.selectWindow(duplicated_image)
            new_image = w.getImage(duplicated_image)
            ij.py.run_macro(f'run("Set Scale...", "distance={distance}␣
↪known={known_scale} unit={unit}");')
            ij.IJ.setRawThreshold(new_image, threshold_low, threshold_high)
            ij.IJ.run(set_string, measure_string)
            for index, row in df.iterrows():
                if row["bacterial_volume"] > size_limit:
                    rm.select(index)
                    ij.IJ.run("Measure")
                    cell_name = f"{channel_name}_{corename}_{index}"
                    cell_list.append(cell_name)
            output_path = f"{channels_path}{corename2}_number.csv"
            ij.IJ.saveAs("Results", output_path)

            #Getting the numbers
            norm_raw = []
            df_number = pandas.read_csv(output_path)
            for i in range(len(df_number)):
                ratio = df_number['RawIntDen'][i] / df_number['Area'][i]
                norm_raw.append(ratio)
            df_number['RawIntDen/Area'] = norm_raw
            df_cell = pandas.DataFrame()
            df_cell['cell_ID'] = cell_list
            df_cell['RawIntDen/Area'] = df_number['RawIntDen/Area']
            output_path2 =␣
↪f"{channels_path}{channel_name}_{corename}_final_number.csv"
            df_cell.to_csv(output_path2)

            #clearing the place
            ij.py.run_macro('close("*");')
            ij.IJ.run("Clear Results")
        rm.runCommand("Reset")

def df_cleaning(df):
    df_transposed = df.transpose()
    df_cleaned = df_transposed.drop(["Unnamed: 0","circle"])
    df_cleaned.rename(columns={df_cleaned.columns[0]: 'd0',
                               df_cleaned.columns[1]: 'd1',
                               df_cleaned.columns[2]: 'd2',
                               df_cleaned.columns[3]: 'd3',
                               df_cleaned.columns[4]: 'd4',
                               df_cleaned.columns[5]: 'd5'}, inplace=True)
    df_cleaned_sorted = df_cleaned.sort_index()
    df_norm = df_cleaned_sorted.sub(df_cleaned_sorted.mean(axis=1), axis=0)
```

```
    df_norm.head()
    for idx, row in df_norm.iterrows():
        plt.plot(range(len(row)),row)
    plt.xlabel('distance (pixels)')
    plt.ylabel('RawIntDen / Area')
    #plt.legend()
    result = f"{directory_path}plot_gal3.svg"
    plt.savefig(result)
    plt.show()

    return df_norm, df_cleaned_sorted


def curves_clustering(df_norm, cluster_number = 10):
    clustering = AgglomerativeClustering(n_clusters=cluster_number)
    clustering.fit(df_norm)
    experiment_labels = clustering.labels_
    df_norm['cluster'] = experiment_labels
    # Print the labels
    #print(experiment_labels)
    for cluster_num in df_norm['cluster'].unique():
        # Filter data for the current cluster
        cluster_data = df_norm[df_norm['cluster'] == cluster_num]

        # Plot the data for the current cluster
        plt.figure(figsize=(8, 6))
        for idx, row in cluster_data.iterrows():
            plt.plot(range(len(row)-1), row[:-1], label=idx)  # Exclude the␣
↪last column 'cluster' from plotting
        plt.title(f'Cluster {cluster_num} Data')
        plt.xlabel('distance (pixels)')
        plt.ylabel('RawIntDen / Area')
        result = f"{directory_path}plot_{cluster_num}.svg"
        plt.savefig(result)        #plt.legend()
        plt.show()
```

[ ]:

```
#define your parent directory
directory_path = '/Users/MrWor/Documents/imaging/20240808 thplc3 h37 gal3 osbp␣
↪alix_sc chmp4b/osbp/6h/'

#Creation of different directory for outputs
bact_path = directory_path + "bact/"
if not os.path.exists(bact_path):
    os.makedirs(bact_path)
measurement_path = directory_path + "measurement/"
if not os.path.exists(measurement_path):
    os.makedirs(measurement_path)
```

```
channels_path = directory_path + "channels/"
if not os.path.exists(channels_path):
    os.makedirs(channels_path)
voronoi_path = directory_path + "voronoi/"
if not os.path.exists(voronoi_path):
    os.makedirs(voronoi_path)
single_cells_path = directory_path + "single_cells/"
if not os.path.exists(single_cells_path):
    os.makedirs(single_cells_path)
grouped_SC = directory_path + "grouped_SC/"
if not os.path.exists(grouped_SC):
    os.makedirs(grouped_SC)
```

0.0.4 this part is to get the different sub-stacks from the raw files useful for later analysis

[ ]:
```
format = f'Tiff'
file_pattern = os.path.join(directory_path, "*.czi")
file_list = glob.glob(file_pattern)
for file_path in file_list:
    raw_image = image = ij.io().open(file_path)
    basename = os.path.basename(file_path)
    corename = os.path.splitext(basename)[0]
    LC3_channel = 2
    channel = raw_image[:, :, LC3_channel, :]
    lc3 = ij.py.to_imageplus(channel)
    lc3.setDimensions(1, 11, 1)
    #ij.ui().show(lc3)
    result_path = f"{channels_path}/LC3_channel_{corename}.tif"
    if os.path.exists(result_path):
        os.remove(result_path)
    ij.IJ.saveAs(lc3, "Tiff", ij.py.to_java(result_path))

    gal3_channel = 0
    channel = raw_image[:, :, gal3_channel, :]
    gal3 = ij.py.to_imageplus(channel)
    gal3.setDimensions(1, 11, 1)
    result_path = f"{channels_path}/osbp_channel_{corename}.tif"
    if os.path.exists(result_path):
        os.remove(result_path)
    ij.IJ.saveAs(gal3, "Tiff", ij.py.to_java(result_path))

    bact_channel = 1
    channel = raw_image[:, :, bact_channel, :]
    bact = ij.py.to_imageplus(channel)
    bact.setDimensions(1, 11, 1)
    result_path = f"{bact_path}/bact_channel_{corename}.tif"
```

```
    if os.path.exists(result_path):
        os.remove(result_path)
    ij.IJ.saveAs(bact, "Tiff", ij.py.to_java(result_path))

    method = 'max'
    nuclei_channel = 3
    blue_channel = raw_image[:, :, nuclei_channel,:]
    sigma = 1.5  # Adjust the value of sigma as needed
    smoothed_image = ij.op().run("smooth", blue_channel, sigma)
    imp = ij.py.to_imageplus(smoothed_image) # convert the image into the␣
↪imageplus type object needed for z-projection
    # Z-projection
    z_projector_result = ZProjector.run(imp, method)
    z_collapsed_image = ij.py.from_java(z_projector_result)
    z_collapsed_dataset = ij.py.to_dataset(z_collapsed_image)
    result_path = f"{voronoi_path}/{corename}_max_nuclei_channel.tif"
    if os.path.exists(result_path):
        os.remove(result_path)
    ij.io().save(z_collapsed_dataset, result_path)
    print(f"Saving image {result_path}.")
```

1 Voronoi segmentation to collect single cell background fluorescence

1.0.1 cellpose segmention

This is to collect the nuclei ROIs that will serve as the base for the voronoi segmentation

[ ]:
```
# segmentation using images ending with "nuclei_channel.tif"
file_pattern = os.path.join(voronoi_path, "*nuclei_channel.tif")
file_list = glob.glob(file_pattern)
model = models.CellposeModel(gpu=True, model_type='THP1_nuclei')
for file_path in file_list:
    imgs = io.imread(file_path)
    channels = [[0,0]]
    masks, flows, styles = model.eval(imgs, diameter=None, channels=channels)
    io.save_to_png(imgs, masks, flows, file_path)
```

[ ]:
```
# this block is to obtain the ROIs from the cellpose segmentation step
file_pattern = os.path.join(directory_path, "*.czi")
file_list = glob.glob(file_pattern)
for file_path in file_list:
    f_name = os.path.basename(file_path)
    corename = os.path.splitext(f_name)[0]
    image_path = f"{voronoi_path}{corename}_max_nuclei_channel.tif"
    image_red = ij.io().open(image_path)
    imp = ij.py.to_imageplus(image_red)
```

```
    input_txt = Path(f"{voronoi_path}/{corename}_max_nuclei_channel_cp_outlines.
↪txt")
    txt_fh = open(input_txt, 'r')
    for line in txt_fh:
        xy = line.rstrip().split(",")
        xy_coords = [int(element) for element in xy if element not in '']
        x_coords = [int(element) for element in xy[::2] if element not in '']
        y_coords = [int(element) for element in xy[1::2] if element not in '']
        xcoords_jint = JArray(JInt)(x_coords)
        ycoords_jint = JArray(JInt)(y_coords)
        polygon_roi_instance = scyjava.jimport('ij.gui.PolygonRoi')
        roi_instance = scyjava.jimport('ij.gui.Roi')
        imported_polygon = polygon_roi_instance(xcoords_jint, ycoords_jint,␣
↪len(x_coords), int(roi_instance.POLYGON))
        imp.setRoi(imported_polygon)
        rm.addRoi(imported_polygon)
    ij.py.run_macro("roiManager('Select All');")
    rm.runCommand("Save", f"{voronoi_path}/" + f"{corename}_nuclei.zip")
    ij.py.run_macro("roiManager('Select All');")
    rm.runCommand("Delete")
```

[ ]:
```
# Voronoi segmentation on the nuclei channel
for file_path in file_list:
    f_name = os.path.basename(file_path)
    corename = os.path.splitext(f_name)[0]
    image_path = f"{voronoi_path}{corename}_max_nuclei_channel.tif"
    image= ij.io().open(image_path)
    input_ROI = f"{voronoi_path}{corename}_nuclei.zip"
    rm.open(input_ROI)
    ij.ui().show(image)
    #to better draw, apply and save ROIs, the image should be displayed:
    imp = ij.py.to_imageplus(image)
    roi_count = rm.getCount()
    for i in range(roi_count):
        rm.select(i)
        shrinking = ij.IJ.run("Enlarge...", "enlarge=-1 pixel")
        rm.runCommand("update")
    rm.runCommand("Select All")
    rm.runCommand("XOR")
    rm.runCommand("Fill")
    rm.runCommand("Select All")
    rm.runCommand("XOR")
    ij.IJ.run("Clear Outside")
    rm.runCommand("Select All")
    rm.runCommand("Delete")
    ij.py.run_macro('setAutoThreshold("Default dark no-reset");')
    ij.IJ.run("Threshold...")
```

```
    ij.py.run_macro('setThreshold(5, 255);')
    ij.py.run_macro('setOption("BlackBackground", true);')
    ij.IJ.run("Convert to Mask", "black")

    voronoi = """
run("Set Measurements...", "center redirect=None decimal=1");
run("Analyze Particles...","size=3-Infinity display clear");
//Resolution de l'image pwidth et pheight
getPixelSize(unit, pw, ph, pd);
//Voronio
run("Voronoi");
setThreshold(0, 0,"black & white");

//Wand to ROI Manager
x=newArray(nResults);
y=newArray(nResults);
nbPoints=nResults;

for (i=0; i<nbPoints; i++) {
    x[i]=getResult("XM",i)/pw;
    y[i]=getResult("YM",i)/ph;
}
for (i=0; i<nbPoints; i++) {
    doWand(x[i], y[i], 156.0, "Legacy");
    roiManager("Add");
}

//Center of mass
/*
x=newArray(nResults);
y=newArray(nResults);
for (i=0; i<nResults; i++) {
    x[i]=getResult("XM",i)/pw;
    y[i]=getResult("YM",i)/ph;
}
*/
makeSelection("point", x, y);
selectWindow("Results");
run("Close");
close("*");
"""
    run_voronoi = ij.py.run_macro(voronoi)
    ij.py.run_macro("roiManager('Select All');")
    rm.runCommand("Save", f"{voronoi_path}/" + f"{corename}_voronoi.zip")
    rm.runCommand("Delete")
```

2 getting single infected cells

[ ]:
```
# variables needed for the future steps.
# The threshold values concerns the bacterial channel for their detection. the␣
↪'low' and 'high' value must be determined in advance, easier done in fiji␣
↪and the
# adjust threshold function.
# The threshold size is the size limit from which the bacteria will be detected
threshold_low = 10
threshold_high = 255
threshold_size = 0.1
# these values are to set the scale of the images
distance = 1024
known = 224.48
unit = "µm"
```

[ ]:
```
get_bacteria_area(bact_path, directory_path, threshold_low, threshold_high,␣
↪distance, known, unit)
```

[ ]:
```
slice_number = 11 # Change this to the actual slice number in your image
slice_distance = 1  # Change this to the actual distance between your slices.␣
↪The unit here is in micrometers
#file_pattern = os.path.join(directory_path, "*area_summary.csv")
file_pattern = os.path.join(directory_path, "*.czi")
file_list = glob.glob(file_pattern)
final_results_path = f"{directory_path}final_results/"
if not os.path.exists(final_results_path):
    os.makedirs(final_results_path)
for file_path in file_list:
    # Read the DataFrame.
    basename = os.path.basename(file_path)
    corename = os.path.splitext(basename)[0]
    csv_path = f"{bact_path}{corename}_area_summary.csv"
    df = pandas.read_csv(csv_path)

    # Calculate the sum of the "Total Area" for each pattern of the "Slice"␣
↪column.
    df1 = calculate_total_volume_per_cell(df)

    #f_name = os.path.splitext(f_name)[0]
    output_path = f"{final_results_path}/{corename}_burden.csv"
    df1.to_csv(output_path)
```

2.0.1 Single cell isolation

This will duplicate every infected cells (or showing a volume of bacteria above the 'size_limit')

[ ]:
```
# enter the names of the channel of interests
cois = ['bact_channel','osbp_channel','LC3_channel']

#function to isolate single cell signals
w = scyjava.jimport('ij.WindowManager')
single_cell_isolation(cois, directory_path, bact_path, voronoi_path,␣
↪channels_path, size_limit = 0.1)
```

3 Single cell fluorescence measurement

this step is to collect the fluorescence intensity in the cells, that can serve as a background value to substrack to the MCV fluorescence. This could eventually improve the classification of bacteria. But this step is not mandatory and the numbers should be compared between the raw and substracted fluorescence bacteria.

[ ]:
```
threshold_low = 1
threshold_high = 255
distance = 1024
known_scale = 224.48
unit = "µm"
cois = ['osbp_channel','LC3_channel']
w = scyjava.jimport('ij.WindowManager')
single_cell_fluorescence(cois, directory_path, voronoi_path, channels_path,␣
↪distance, known_scale, unit, size_limit = 0.1)
```

4 single cell bacteria detection and measurement

[ ]:
```
#Trackmate settings

dsettings = { # specify parameters for the detection step in Trackmate␣
↪(Threshold Detector)
    'TARGET_CHANNEL' : ij.py.to_java(1),
    'SIMPLIFY_CONTOURS' : False,
    'INTENSITY_THRESHOLD' : 10.0,
}
quality_filter = 50
tsettings = { # specify parameters for the tracking step in Trackmate (LAP␣
↪Tracker)
    'LINKING_MAX_DISTANCE' : 10.0,
    'ALLOW_GAP_CLOSING' : True,
    'GAP_CLOSING_MAX_DISTANCE' : 4.0,
    'MAX_FRAME_GAP' : ij.py.to_java(2),
    'ALLOW_TRACK_SPLITTING' : False,
    'SPLITTING_MAX_DISTANCE' : 15.0,
    'ALLOW_TRACK_MERGING' : False,
}
```

```
[ ]: threshold_low = 10
threshold_high = 255
threshold_size = 0.1
distance = 1024
known = 224.48
unit = "µm"
file_pattern = os.path.join(directory_path, "*.czi")
file_list = glob.glob(file_pattern)
for file_path in file_list:
    basename = os.path.basename(file_path)
    corename = os.path.splitext(basename)[0]
    single_cells_image_path = f"{single_cells_path}{corename}/"
    get_bact_rois(single_cells_image_path) # Collect Bacteria ROIs using␣
 ↪particle detection of Fiji
    track_bacteria(directory_path, single_cells_image_path, dsettings,␣
 ↪quality_filter, tsettings, display=False) # detect bacteria centroid using␣
 ↪trackmate
    merge_trackmate_rois(single_cells_image_path, directory_path, grouped_SC) #␣
 ↪Assigment of a single ROI from the pool in the first step to the centroid␣
 ↪detected in trackmate
    apply_bact_overlays(single_cells_image_path, grouped_SC)# Overla
```

4.0.1 Saving ROIs in a dedicated folder

```
[ ]: file_pattern = os.path.join(directory_path, "*.czi")
file_list = glob.glob(file_pattern)
for file_path in file_list:
    basename = os.path.basename(file_path)
    corename_image = os.path.splitext(basename)[0]
    single_cells_image_path = f"{single_cells_path}{corename_image}/"
    saving_rois(single_cells_image_path)
```

4.0.2 circle measurement around bacteria in single cells

```
[ ]: distance = 1024
known_scale = 224.48
unit = "µm"
file_pattern = os.path.join(directory_path, "*.czi")
file_list = glob.glob(file_pattern)

# enters the channels in your image
cois = ['osbp_channel', 'LC3_channel', 'bact_channel']
bact_channel = cois[2]
set_string = f'Set Measurements...'
measure_string = f'mean area integrated stack redirect=None decimal=2'
ij.IJ.run(set_string, measure_string)
for file_path in file_list:
```

```
    basename = os.path.basename(file_path)
    corename = os.path.splitext(basename)[0]
    single_cells_image_path = f"{single_cells_path}{corename}/"
    file_pattern2 = os.path.join(single_cells_image_path, "*.tif")
    file_list2 = glob.glob(file_pattern2)
    for image in file_list2:
        if not image.endswith('overlay.tif'):
            basename2 = os.path.basename(image)
            corename2 = os.path.splitext(basename2)[0]
            if corename2.startswith(cois[0]):
                channel = cois[0]
                f_name = corename2.split(f"{channel}_")[1]
                bact_roi_path = f"{single_cells_image_path}rois_bact/{f_name}"
                imp = ij.IJ.openImage(image)
                print(f"opening {corename2}")
                imp.show()
                ij.IJ.run(imp, "Select None", "")
                circlesofhell(bact_roi_path, channel, distance, known_scale,␣
↪unit, imp) # This is the circle workflow
                ij.py.run_macro('close("*");')
            elif corename2.startswith(cois[1]):
                channel = cois[1]
                f_name = corename2.split(f"{channel}_")[1]
                bact_roi_path = f"{single_cells_image_path}rois_bact/{f_name}"
                imp = ij.IJ.openImage(image)
                print(f"opening {corename2}")
                imp.show()
                ij.IJ.run(imp, "Select None", "")
                circlesofhell(bact_roi_path, channel, distance, known_scale,␣
↪unit, imp)
                ij.py.run_macro('close("*");')
```

5 concatenation of CSVs

[ ]:
```
file_pattern = os.path.join(directory_path, "*.czi")
file_list = glob.glob(file_pattern)
df_lc3_list = []
df_gal3_list = []

df_lc3 = pandas.DataFrame({'circle': [1, 2, 3, 4, 5, 6]})
df_lc3_list.append(df_lc3['circle'])
df_gal3 = pandas.DataFrame({'circle': [1, 2, 3, 4, 5, 6]})
df_gal3_list.append(df_lc3['circle'])

for file_path in file_list:
    basename = os.path.basename(file_path)
```

```
    corename = os.path.splitext(basename)[0]
    bact_roi_path = f"{single_cells_path}{corename}/rois_bact/"
    for cells in os.listdir(bact_roi_path):
        csv_path = f"{bact_roi_path}{cells}/"
        file_pattern2 = os.path.join(csv_path, "*.csv")
        file_list2 = glob.glob(file_pattern2)
        for csv in file_list2:
            if csv.endswith('LC3_channel.csv'):
                df= pandas.read_csv(csv)
                norm_raw = []
                basename = os.path.basename(csv)
                roi_number = basename.split("_")[0]
                cell_name = f"{cells}_{roi_number}"
                for i in range(len(df)):
                    ratio = df['RawIntDen'][i] / df['Area'][i]
                    norm_raw.append(ratio)
                df[cell_name] = norm_raw
                df_lc3_list.append(df[cell_name])

            if csv.endswith('osbp_channel.csv'):
                df= pandas.read_csv(csv)
                norm_raw = []
                basename = os.path.basename(csv)
                roi_number = basename.split("_")[0]
                cell_name = f"{cells}_{roi_number}"
                for i in range(len(df)):
                    ratio = df['RawIntDen'][i] / df['Area'][i]
                    norm_raw.append(ratio)
                df[cell_name] = norm_raw
                df_gal3_list.append(df[cell_name])

    result_lc3 = pandas.concat(df_lc3_list, axis=1)
    result_gal3 = pandas.concat(df_gal3_list, axis=1)
    result_lc3.to_csv(f"{directory_path}LC3_numbers_Rawint_area.csv")
    result_gal3.to_csv(f"{directory_path}osbp_numbers_Rawint_area.csv")
```

[ ]:
```
file_pattern = os.path.join(directory_path, "*.czi")
file_list = glob.glob(file_pattern)
df_lc3_list = []
df_gal3_list = []

df_lc3 = pandas.DataFrame({'circle': [1, 2, 3, 4, 5, 6]})
df_lc3_list.append(df_lc3['circle'])
df_gal3 = pandas.DataFrame({'circle': [1, 2, 3, 4, 5, 6]})
df_gal3_list.append(df_lc3['circle'])

for file_path in file_list:
```

```
    basename = os.path.basename(file_path)
    corename = os.path.splitext(basename)[0]
    bact_roi_path = f"{single_cells_path}{corename}/rois_bact/"
    for cells in os.listdir(bact_roi_path):
        csv_path = f"{bact_roi_path}{cells}/"
        file_pattern2 = os.path.join(csv_path, "*.csv")
        file_list2 = glob.glob(file_pattern2)
        for csv in file_list2:
            if csv.endswith('LC3_channel.csv'):
                df= pandas.read_csv(csv)
                norm_raw = []
                basename = os.path.basename(csv)
                roi_number = basename.split("_")[0]
                cell_name = f"{cells}_{roi_number}"
                for i in range(len(df)):
                    prout = df['Mean'][i]
                    norm_raw.append(prout)
                df[cell_name] = norm_raw
                df_lc3_list.append(df[cell_name])

            if csv.endswith('osbp_channel.csv'):
                df= pandas.read_csv(csv)
                norm_raw = []
                basename = os.path.basename(csv)
                roi_number = basename.split("_")[0]
                cell_name = f"{cells}_{roi_number}"
                for i in range(len(df)):
                    prout = df['Mean'][i]
                    norm_raw.append(prout)
                df[cell_name] = norm_raw
                df_gal3_list.append(df[cell_name])

    result_lc3 = pandas.concat(df_lc3_list, axis=1)
    result_gal3 = pandas.concat(df_gal3_list, axis=1)
    result_lc3.to_csv(f"{directory_path}LC3_numbers_MFI.csv")
    result_gal3.to_csv(f"{directory_path}osbp_numbers_MFI.csv")
```

5.0.1 this one below is to use only on Gal3 to substract the average value of each cell

[ ]:
```
file_pattern = os.path.join(directory_path, "*.czi")
file_list = glob.glob(file_pattern)
df_lc3_list = []
df_gal3_list = []

df_lc3 = pandas.DataFrame({'circle': [1, 2, 3, 4, 5, 6]})
df_lc3_list.append(df_lc3['circle'])
df_gal3 = pandas.DataFrame({'circle': [1, 2, 3, 4, 5, 6]})
```

```
df_gal3_list.append(df_lc3['circle'])

for file_path in file_list:
    basename = os.path.basename(file_path)
    corename = os.path.splitext(basename)[0]
    csv_path = f"{channels_path}gal3_channel_{corename}_final_number.csv"
    df = pandas.read_csv(csv_path)
    bact_roi_path = f"{single_cells_path}{corename}/rois_bact/"
    cell_list = os.listdir(bact_roi_path)
    for i in range(len(df)):
        cell_name = df['cell_ID'][i]
        gauche = cell_name.split("_")[2]
        droite = cell_name.split("_")[3]
        folder_name = f"{gauche}_{droite}"
        folder_path = f"{bact_roi_path}{folder_name}/"
        csv_pattern  = os.path.join(folder_path, "*gal3_channel.csv")
        csv_list = glob.glob(csv_pattern)
        for csv in csv_list:
            df2 = pandas.read_csv(csv)
            basename = os.path.basename(csv)
            roi_number = basename.split("_")[0]
            cell_name = f"{folder_name}_{roi_number}"
            norm_raw = []
            for n in range(len(df2)):
                ratio = df2['RawIntDen'][n] / df2['Area'][n]
                norm_ratio = ratio - df['RawIntDen/Area'][i]
                norm_raw.append(norm_ratio)
            df2[cell_name] = norm_raw
            df_gal3_list.append(df2[cell_name])

    result_gal3 = pandas.concat(df_gal3_list, axis=1)
    result_gal3.to_csv(f"{directory_path}gal3_numbers_norm.csv")
```

[ ]:
```
csv_path = f"{channels_path}gal3_channel_Experiment-2168_final_number.csv"
df = pandas.read_csv(csv_path)
df.head()
```

[ ]:
```
prout = df['cell_ID'][1]
fessegauche = prout.split("_")[2]
fessedroite = prout.split("_")[3]
fesses = f"{fessegauche}_{fessedroite}"
print(fesses)
```

6 saving a montage of each detected phagosome for visual curation of the clustering

[ ]:
```
cois = ['bact_channel', 'osbp_channel','LC3_channel']
w = scyjava.jimport('ij.WindowManager')
single_composite_isolation(cois, directory_path, voronoi_path, size_limit = 0.1)
```

[ ]:
```
# save a montage of each MCV
file_pattern = os.path.join(directory_path, "*.czi")
file_list = glob.glob(file_pattern)
w = scyjava.jimport('ij.WindowManager')
for file_path in file_list:
    basename = os.path.basename(file_path)
    corename = os.path.splitext(basename)[0]
    bact_roi_path = f"{single_cells_path}{corename}/rois_bact/"
    for folder in os.listdir(bact_roi_path):
        image_folder = f"{bact_roi_path}/{folder}/"
        image_path = f"{image_folder}{folder}.tif"
        imp = ij.IJ.openImage(image_path)
        imp.show()
        imp.setDisplayMode(1)
        imp.setC(1)
        ij.IJ.run(imp, "Red", "")
        ij.py.run_macro("setMinAndMax(0, 100);")
        imp.setC(2)
        ij.IJ.run(imp, "Blue", "")
        ij.py.run_macro("setMinAndMax(0, 100);")
        imp.setC(3)
        ij.IJ.run(imp, "Green", "")
        ij.py.run_macro("setMinAndMax(0, 100);")
        imp.setC(4)
        ij.IJ.run(imp, "Grays", "")
        #ij.IJ.run(imp, "RGB Color", "slices")
        roi_pattern = os.path.join(image_folder, "*.roi")
        roi_list = glob.glob(roi_pattern)
        for roi in roi_list:
            roi_basename = os.path.basename(roi)
            roi_corename = os.path.splitext(roi_basename)[0]
            open_roi = rm.open(roi)
            roi = rm.getRoi(0)
            selected = rm.select(0)
            x = roi.getContourCentroid()[0]
            y = roi.getContourCentroid()[1]
            square = imp.setRoi(int(x-20), int(y-20), 40, 40)
            added = rm.addRoi(square)
            roi2 = rm.getRoi(1)
            roi3 = rm.select(1)
```

```
            square_name = f"{folder}_{roi_corename}"
            ij.IJ.run("Duplicate...", f"title={str(square_name)}")
            new_vid =  w.getImage(str(square_name))
            ij.IJ.run(new_vid, "Make Montage...", "columns=4 rows=1 scale=2␣
↪border=1")
            new_montage =  w.getImage("Montage")
            output_path = f"{measurement_path}{square_name}.tif"
            if os.path.exists(output_path):
                os.remove(output_path)
            ij.IJ.save(new_montage, output_path)
            rm.reset()
            w.getImage("Montage").close()
            w.getImage(str(square_name)).close()
            ij.IJ.selectWindow(imp.getTitle())
        ij.py.run_macro('close("*");')
```

7 Classification of the stainings

[ ]:
```
csv_lc3 = f"{directory_path}LC3_numbers_Rawint_area.csv"
csv_gal3 = f"{directory_path}osbp_numbers_Rawint_area.csv"
df_lc3 = pandas.read_csv(csv_lc3)
df_gal3 = pandas.read_csv(csv_gal3)
```

[ ]:
```
# Cleaning LC3 signals
df_lc3_norm, df_lc3_cleaned_sorted = df_cleaning(df_lc3)
```

[ ]:
```
#clustering the signal using sklearn clustering method
clustering = curves_clustering(df_lc3_norm, cluster_number = 10)
```

7.0.1 Visualization of specific cluster in scrollable window

[ ]:
```
# this is to define which cluster to vizualize. It will grab the montage images␣
↪for the designated cluster group and display them.
image_list = []
cluster_num = 5
for i in range(len(df_lc3_norm)):
    if df_lc3_norm['cluster'][i] == cluster_num:
        image = df_lc3_norm.index[i]
        image_path = Path(f"{measurement_path}{image}.tif").as_posix()
        image_list.append(image_path)
```

[ ]:
```
# This is how to display the images bound to the cluster defined above.
# Still need to find a way to open this as a separated window that does not␣
↪block the whole session so we can display multiple windows with multiple␣
↪outputs.
class MainWindow(QMainWindow):
```

```
    def __init__(self, image_list):
        super().__init__()
        self.image_list = image_list
        self.window()

    def window(self):
        self.scroll = QScrollArea()
        self.win = QWidget()
        self.grid = QGridLayout()

        num_images = len(self.image_list)
        num_columns = 4  # Fixed number of columns
        num_rows = (num_images + num_columns - 1) // num_columns  # Calculate␣
↪number of rows dynamically

        n = 0
        for i in range(num_rows):
            for j in range(num_columns):
                if n < num_images:
                    file = self.image_list[n]
                    pixmap = QPixmap(file)
                    pixmap2 = pixmap.scaled(500, 200, Qt.KeepAspectRatio)
                    label = QLabel(pixmap=pixmap2)
                    self.grid.addWidget(label, i, j)
                    n += 1

        self.win.setLayout(self.grid)
        self.scroll.setWidgetResizable(True)
        self.scroll.setVerticalScrollBarPolicy(Qt.ScrollBarAlwaysOn)
        self.scroll.setHorizontalScrollBarPolicy(Qt.ScrollBarAlwaysOn)
        self.scroll.setWidget(self.win)
        self.setCentralWidget(self.scroll)
        self.setGeometry(0, 0, 2300, 1000)
        self.setWindowTitle('Image Viewer')
        self.show()

def main():
    app = QApplication(sys.argv)
    main = MainWindow(image_list)
    sys.exit(app.exec_())

if __name__ == '__main__':
    main()
```

[ ]:
```
clusters_positives_lc3 = [5, 4, 2, 8, 0, 9, 7, 6]
clusters_negatives_lc3 = [3, 1]
```

```
df_lc3_norm.loc[df_lc3_norm['cluster'].isin(clusters_positives_lc3), 'cluster']␣
↪= 10
df_lc3_norm.loc[df_lc3_norm['cluster'].isin(clusters_negatives_lc3), 'cluster']␣
↪= 11
df_lc3_norm.head()
```

[ ]:
```
df_cluster_3 = df_lc3_norm[df_lc3_norm['cluster'] == 4].
↪drop(columns=['cluster'])
df_cluster_3.head()
```

[ ]:
```
# Run the clustering function on the filtered data
clustering = curves_clustering(df_cluster_3, cluster_number = 3)
```

[ ]:
```
image_list = []
cluster_num = 2
for i in range(len(df_cluster_3)):
    if df_cluster_3['cluster'][i] == cluster_num:
        image = df_cluster_3.index[i]
        image_path = Path(f"{measurement_path}{image}.tif").as_posix()
        image_list.append(image_path)
```

[ ]:
```
class MainWindow(QMainWindow):

    def __init__(self, image_list):
        super().__init__()
        self.image_list = image_list
        self.window()

    def window(self):
        self.scroll = QScrollArea()
        self.win = QWidget()
        self.grid = QGridLayout()

        num_images = len(self.image_list)
        num_columns = 4  # Fixed number of columns
        num_rows = (num_images + num_columns - 1) // num_columns  # Calculate␣
↪number of rows dynamically

        n = 0
        for i in range(num_rows):
            for j in range(num_columns):
                if n < num_images:
                    file = self.image_list[n]
                    pixmap = QPixmap(file)
                    pixmap2 = pixmap.scaled(500, 200, Qt.KeepAspectRatio)
                    label = QLabel(pixmap=pixmap2)
                    self.grid.addWidget(label, i, j)
```

```
                    n += 1

        self.win.setLayout(self.grid)
        self.scroll.setWidgetResizable(True)
        self.scroll.setVerticalScrollBarPolicy(Qt.ScrollBarAlwaysOn)
        self.scroll.setHorizontalScrollBarPolicy(Qt.ScrollBarAlwaysOn)
        self.scroll.setWidget(self.win)
        self.setCentralWidget(self.scroll)
        self.setGeometry(0, 0, 2300, 1000)
        self.setWindowTitle('Image Viewer')
        self.show()

def main():
    app = QApplication(sys.argv)
    main = MainWindow(image_list)
    sys.exit(app.exec_())

if __name__ == '__main__':
    main()
```

[ ]:
```
clusters_positives_lc3_2 = [1]
clusters_negatives_lc3_2 = [0, 2]
df_cluster_3.loc[df_cluster_3['cluster'].isin(clusters_positives_lc3_2),␣
 ↪'cluster'] = 10
df_cluster_3.loc[df_cluster_3['cluster'].isin(clusters_negatives_lc3_2),␣
 ↪'cluster'] = 11
```

[ ]:
```
df_cluster_3.rename(columns={'cluster': 'new_cluster'}, inplace=True)

# Update the original dataframe with the new cluster labels for cluster 4
df_lc3_norm.loc[df_lc3_norm['cluster'] == 4, 'cluster'] =␣
 ↪df_cluster_3['new_cluster'].values
#df_gal3_norm.to_csv(f"{directory_path}df_gal3_norm.csv")
```

7.0.2 same workflow for the second marker

[ ]:
```
df_gal3_norm, df_gal3_cleaned_sorted = df_cleaning(df_gal3)
clustering = curves_clustering(df_gal3_norm, cluster_number = 10)
```

[ ]:
```
image_list = []
cluster_num = 4
for i in range(len(df_gal3_norm)):
    if df_gal3_norm['cluster'][i] == cluster_num:
        image = df_gal3_norm.index[i]
        image_path = Path(f"{measurement_path}{image}.tif").as_posix()
        image_list.append(image_path)
```

[ ]:
```python
class MainWindow(QMainWindow):

    def __init__(self, image_list):
        super().__init__()
        self.image_list = image_list
        self.window()

    def window(self):
        self.scroll = QScrollArea()
        self.win = QWidget()
        self.grid = QGridLayout()

        num_images = len(self.image_list)
        num_columns = 4  # Fixed number of columns
        num_rows = (num_images + num_columns - 1) // num_columns  # Calculate␣
 ↪number of rows dynamically

        n = 0
        for i in range(num_rows):
            for j in range(num_columns):
                if n < num_images:
                    file = self.image_list[n]
                    pixmap = QPixmap(file)
                    pixmap2 = pixmap.scaled(500, 200, Qt.KeepAspectRatio)
                    label = QLabel(pixmap=pixmap2)
                    self.grid.addWidget(label, i, j)
                    n += 1

        self.win.setLayout(self.grid)
        self.scroll.setWidgetResizable(True)
        self.scroll.setVerticalScrollBarPolicy(Qt.ScrollBarAlwaysOn)
        self.scroll.setHorizontalScrollBarPolicy(Qt.ScrollBarAlwaysOn)
        self.scroll.setWidget(self.win)
        self.setCentralWidget(self.scroll)
        self.setGeometry(0, 0, 2300, 1000)
        self.setWindowTitle('Image Viewer')
        self.show()

def main():
    app = QApplication(sys.argv)
    main = MainWindow(image_list)
    sys.exit(app.exec_())

if __name__ == '__main__':
    main()
```

7.0.3 Reclustering insatisfying groups

```
[ ]: clusters_positives_gal3 = [2, 1, 4, 6, 7, 5]
     clusters_negatives_gal3 = [3, 0, 9, 8]
     df_gal3_norm.loc[df_gal3_norm['cluster'].isin(clusters_positives_gal3),␣
      ↪'cluster'] = 10
     df_gal3_norm.loc[df_gal3_norm['cluster'].isin(clusters_negatives_gal3),␣
      ↪'cluster'] = 11
     df_gal3_norm.head()
```

```
[ ]: df_cluster_3 = df_gal3_norm[df_gal3_norm['cluster'] == 1].
      ↪drop(columns=['cluster'])
     df_cluster_3.head()
```

```
[ ]: # Run the clustering function on the filtered data
     clustering = curves_clustering(df_cluster_3, cluster_number = 3)
```

```
[ ]: image_list = []
     cluster_num = 0
     for i in range(len(df_cluster_3)):
         if df_cluster_3['cluster'][i] == cluster_num:
             image = df_cluster_3.index[i]
             image_path = Path(f"{measurement_path}{image}.tif").as_posix()
             image_list.append(image_path)
```

```
[ ]: class MainWindow(QMainWindow):

         def __init__(self, image_list):
             super().__init__()
             self.image_list = image_list
             self.window()

         def window(self):
             self.scroll = QScrollArea()
             self.win = QWidget()
             self.grid = QGridLayout()

             num_images = len(self.image_list)
             num_columns = 4  # Fixed number of columns
             num_rows = (num_images + num_columns - 1) // num_columns  # Calculate␣
      ↪number of rows dynamically

             n = 0
             for i in range(num_rows):
                 for j in range(num_columns):
                     if n < num_images:
                         file = self.image_list[n]
```

```
                    pixmap = QPixmap(file)
                    pixmap2 = pixmap.scaled(500, 200, Qt.KeepAspectRatio)
                    label = QLabel(pixmap=pixmap2)
                    self.grid.addWidget(label, i, j)
                    n += 1

        self.win.setLayout(self.grid)
        self.scroll.setWidgetResizable(True)
        self.scroll.setVerticalScrollBarPolicy(Qt.ScrollBarAlwaysOn)
        self.scroll.setHorizontalScrollBarPolicy(Qt.ScrollBarAlwaysOn)
        self.scroll.setWidget(self.win)
        self.setCentralWidget(self.scroll)
        self.setGeometry(0, 0, 2300, 1000)
        self.setWindowTitle('Image Viewer')
        self.show()

def main():
    app = QApplication(sys.argv)
    main = MainWindow(image_list)
    sys.exit(app.exec_())

if __name__ == '__main__':
    main()
```

[ ]:
```
clusters_positives_gal3_2 = [1]
clusters_negatives_gal3_2 = [0, 2]
df_cluster_3.loc[df_cluster_3['cluster'].isin(clusters_positives_gal3_2),␣
 ↪'cluster'] = 10
df_cluster_3.loc[df_cluster_3['cluster'].isin(clusters_negatives_gal3_2),␣
 ↪'cluster'] = 11
```

[ ]:
```
df_cluster_3.rename(columns={'cluster': 'new_cluster'}, inplace=True)

# Update the original dataframe with the new cluster labels for cluster 4
df_gal3_norm.loc[df_gal3_norm['cluster'] == 1, 'cluster'] =␣
 ↪df_cluster_3['new_cluster'].values
#df_gal3_norm.to_csv(f"{directory_path}df_gal3_norm.csv")
```

[ ]:
```
df_gal3_norm.head()
```

[ ]:
```
print(df_gal3_norm)
```

7.0.4 Quantification of the frequency of "positive" vs "negative" phagosomes for the different markers

[ ]:
```
# Enter the cluster number matching each category
clusters_positives_lc3 = [10]
clusters_negatives_lc3 = [11]
clusters_positives_gal3 = [10]
clusters_negatives_gal3 = [11]
```

[ ]:
```
# Classify each phagosome
lc3_positive = []
gal3_positive = []
double_positive = []
negatives = []

for i in range(len(df_lc3_norm)):
    image = df_lc3_norm.index[i]
    if df_lc3_norm['cluster'][i] in clusters_positives_lc3 and␣
↪df_gal3_norm['cluster'][i] in clusters_positives_gal3:
        double_positive.append(image)
    elif df_lc3_norm['cluster'][i] in clusters_positives_lc3 and␣
↪df_gal3_norm['cluster'][i] in clusters_negatives_gal3:
        lc3_positive.append(image)

    elif df_lc3_norm['cluster'][i] in clusters_negatives_lc3 and␣
↪df_gal3_norm['cluster'][i] in clusters_positives_gal3:
        gal3_positive.append(image)
    else:
        negatives.append(image)
```

[ ]:
```
#Creation of a table summarizing each category
total_images = len(df_lc3_norm)
df = pandas.DataFrame({
    'category': ['lc3_positive', 'osbp_positive', 'double_positive',␣
↪'negatives'],
    'count': [len(lc3_positive), len(gal3_positive), len(double_positive),␣
↪len(negatives)]
})
df['% total'] = (df['count'] / total_images) * 100
df.head()
```

[ ]:
```
df_gal3_norm.to_csv(f"{directory_path}df_gal3_norm.csv")
```

[ ]:
```
#export of the table
df.to_csv(f"{directory_path}Lc3_osbp_%quantif.csv")
```

[ ]:
```
```

[ ]:

```

```

[ ]:

```
df_cluster_4 = df_gal_norm[df_gal_norm['cluster'] == 4].
 ↪drop(columns=['cluster'])

# Run the clustering function on the filtered data
curves_clustering(df_cluster_4, cluster_number = 3)  # Adjust the number of␣
 ↪clusters
df_cluster_4['new_cluster'] = clustering.fit(df_cluster_4)

# Update the original dataframe with the new cluster labels for cluster 4
df_gal_norm.loc[df_gal_norm['cluster'] == 4, 'cluster'] =␣
 ↪df_cluster_4['new_cluster'].values
```
