# Supplementary material for: Dynamic interplay of autophagy and membrane repair during Mycobacterium tuberculosis Infection
Source: PLoS Pathog. 2025 Jan 2;21(1):e1012830. doi: 10.1371/journal.ppat.1012830 (PMC11731705; doi:10.1371/journal.ppat.1012830)
Supplement: Attachment — Submitted filename: ReviewPPath.pdf [file ppat.1012830.s013.pdf]

We sincerely thank the reviewers for their constructive feedback and insightful suggestions. In response, we have revised the manuscript to address their comments. A major new finding is that we could reproduce our data in primary human monocyte-derived macrophages which substantially increases the relevance of our observation in the human macrophage-like cell line THP-1. Specifically, Figure 4 now includes expanded results in panels C–E, and Figure 5 has been supplemented with new panels F and G. Additionally, Figure 6 was updated with modifications to panels A and B. The supplementary data section has been expanded to include three additional figures (Figures S6, S7, and S8). Efforts were also made to improve the clarity of the main text, including a reorganization of the discussion to enhance readability and coherence.

Below, we provide a detailed, point-by-point responses to each reviewer's comments. We hope these revisions adequately address their concerns and enhance the manuscript.

Reviewer's Responses to Questions

Part II – Major Issues: Key Experiments Required for Acceptance

Reviewer #1: Major Point(s)

First, I would like to thank the authors for making accessible their "single_bact_quant" source code. However, it is not very clear in their manuscript how LC3 positivity was determined and which threshold value was used (Fig.1-Fig.2). Indeed, in Fig.1 & Fig.2, the authors report heterogeneity in LC3 recruitment over time, but information is lacking about how a LC3 positive MCV was determined. What was the fluorescence cut off to determine LC3(+) or LC3(-) MCV? How was it calculated? When looking at Panels 2B-C & 2D-E, the Mean Fluorescence Intensity (MFI) associated to the MCV can range from 15 to 100 MFI and still considered by the authors as positive. Can the authors comment about this? Moreover, adding an extra (dashed/coloured) line on the Y-axis of their graphs (Fig2) to highlight where the positive threshold has been set would be great to facilitate the readers understanding.

We thank the constructive feedback provided. In Figure 1A and 2A, the MCVs were visually scored for the positivity, not by an automated calculation. As for the fig2D-E, these are representative quantification on MCVs that are part of the groups described in fig 2A that were visually scored for positivity. The MFI quantification was only done on the representative MCV and was done using the manual tracking method described here (DOI: 10.1007/978-1-4939-6581-6_11). Even if the fluorescence may appear low in number, for 2E specifically the background signal is stable at around 2 A.U which is much lower than the signal when it was considered positive. Any signal higher than the background would be considered positive in the visual assessment. Indeed, the method was not specifically described in the text, which was modified accordingly:

Line 126: "The LC3 recruitment to the MCV was visually assessed from the time of phagocytosis to the end of recording for individual MCV."

Line 625:" For the quantification of frequency of recruitment in Figure 1, and the assessment and categorization of LC3 recruitment in Figure 2A, the MCV were visually scored as positive or negative. The method for manual tracking and quantifying the fluorescence at the MCV over time in Figure 2A and 5 was previously described."

In Fig.3, the authors claim that there is no correlation between LC3 recruitment, the time post-phagocytosis and the bacterial burden. There are several issues with such claim.

First, the R2 evaluates the scatter of the data points around the fitted regression line, but does not indicate anything about statistical significance (Fig3A). An appropriate p-value should be calculated to determine whether the "no correlation" observed is statistically significant or just random. Could the authors please look into this.

A spearman correlation test was performed and yielded a R = -0.2671 and a *p* value *p* = 0.0315. Thus, even if there is a weak negative correlation, it does not match with the data reported in Figure 3B. In the revised figure, the p-values were reported instead of the "ns" on the graph. The text was also modified to include a statement about the correlation as follow line 164:" A modest negative correlation can be observed between the number of bacteria per MCV and the time of recruitment of LC3 to the MCV (R= -0.2671, *p*=0.0315)."

Second, from the Material and Methods section it seems that the current resolution, is not high enough to allow the authors to identify/segment single-bacterial cell and therefore claiming that the authors can calculate the number of bacteria per MCV seems to be overstated.

Indeed, the authors mention the following statement "The cells were imaged using a Zeiss laser scanning confocal microscope LSM-800, equipped with two gallium arsenide phosphide photomultiplier tube (GaAsP-PMT) detectors and a transmitted light photomultiplier tube detector (T-PMT), using the 63x/NA1.4 Oil objective. The observation chamber was maintained at 37oC and supplied with 5% CO2 through a humidifier system. A 10 µm z-stack (1 µm steps) was acquired every 10 minutes on 4 to 8 fields of view over 16 to 20 hours."

Can the author comment about the X, Y and Z resolution achieved with their microscopy system, eventually by looking at the properties/metadata of their acquired images? Can they really achieve single-bacterial cell segmentation within infected macrophages specially when looking at Z-stack distant from each other of 1µm, and not reconstituting Z-projection? Can they comment about how was this done? To the reviewer's knowledge, achieving automated single-bacterial cell resolution on intracellular mycobacteria has not been reported in the literature so far, and therefore would be a real Tour de Force from the authors. Can you comment on this and provide all the details require to support such conclusion? This is important for the entire community, and people developing innovative quantitative imaging approaches. The use of bacterial area (if analysed in 2D) or volume (if analysed in 3D using maximum projection) expressed in pixel2/µm2 or pixel3/µm3 on the other end seems more appropriate.

We thank the reviewer for pointing this. The methods section could indeed be clearer and in this regard and we made some changes (see below). The pixel size is 0.099µm x 0.099µm, which could in theory be enough to segment single bacteria. However, for figures 3A and 3B, the quantification was done also manually and visually by estimating the number of bacteria per aggregate as previously done. We are aware of how subjected to error this approach can be, and this drove the use of the BBQ workflow in the Figure 3C-I to obtain the more reliable bacterial volume metric but indeed not as absolute as an exact bacilli count. An estimation of the number of bacilli in each MCV could be theoretically calculated from a theoretical single bacillus volume but this value is practically complicated to obtain. We agree that any implementation of such workflow could be helpful not just for our work but was not explored further due to the lack of strong results and complexity of such implementation.

Accordingly, the method section was updated and the following sentences were added:

Lines 597: Each field of view was 1024 x 1024 or 2048 x 2048 pixels corresponding to 101.81 x 101.81µm and 202,83 x 202.83 µm, respectively.

In their Fig4A, the authors first investigate the potential relationship between LC3 recruitment and subcellular acidification, and report that LC3 positivity and Lysotracker Blue positivity are mutually exclusive.

Here is their statement: "Strikingly, at 24 hours post-infection, none of the LC3+ MCVs were positive for LTB, although about 15% of MCVs were positive for LTB (Figure 4B). These results strongly suggested that LC3 recruitment does not result in Mtb contained within autophagolysosome".

However, based on the data presented in Fig4-AB, it seems that the dual-positive event was observed and quantified. In addition, the authors report 15% of LysoTracker positivity in the text, but between 25-30% seem more appropriate based on the graph 4B. As previously, request for LC3, can the author comment on LysoTracker positivity how positivity was defined?

As previously stated, the positivity of markers on the MCVs was visually assessed across the images. The representative images in Figure 4A are examples of the different type of MCVs observed.

To support their claim, regarding the lack of maturation in such compartment, have the authors considered performing some additional control experiments with alternative functional proteolytic probes such as DQ-BSA or BMV109 or even staining for V-ATPase or Cathepsins?

We thank the reviewer for the insightful suggestion, as there could be a difference between the autophagosome maturation and its measurable effect such as acidification. To address this concern, we analyzed the LAMP-1 immunostaining of MCVs and the result is now presented in figure 5. It revealed a frequent staining of the MCV and the LC3 TVS structures with LAMP-1, supporting an actual maturation of the autophagosome in terms of membrane composition and fusion with endosomes/lysosomes. However, in the absence of acidification, likely due to some damage (Figure 6A), this may not translate into a fully functional maturation of the MCV as some proteases are only active at acidic pH. Due to these results, we changed the manuscript to soften our conclusion in regard to MCV maturation and bactericidal properties (Line 485-494).

In Fig4C, it is not clear what the authors wanted to report. "THP-1-LC3-GFP cells were treated with IFN-γ 10ng/mL or left untreated overnight and infected with DsRed expressing H37Rv at MOI 2. Cells were also stained with Lysoview-633 (LV) and the dye was kept during image acquisition. Cells were image by time lapse confocal microscopy". Why the authors do not report each individual channels? It is impossible to see LC3 or LV positive event with such representation. Please could the authors consider an alternative presentation of their images?

By showing the T-PMT channel we wanted to emphasize that the major effect of the IFN-g treatment was an increase in cell death but we understand the point raised by the reviewer. We agree with the reviewer's comment and the images associated with the cell death quantification were moved to the Figure S4. New representative images at 6 h post-infection with the fluorescence channels with the segmented cells outlines were used for the new figure.

It is also not clear why the authors only reported data at 6h post infection, where they have performed live-imaging over 20 hours. Could they add more timepoints, or even considered replotting their data highlighting the dynamics in the system rather than just focusing on one timepoint? How the 6 hours was chosen? Some representation as displayed in Fig5, for both IFN-gamma treated or the control condition, would be more appropriate to illustrate their findings and conclusions.

The 6h post infection time was chosen based on previous literature on IFN-γ treatment where the macrophages were pre-treated with IFN-gamma, infected and analyzed shortly after infection (Korster et al 2017). The 6 h time point was arbitrarily decided as comparable early time point after the infection based on other studies but also advanced enough to quantify the fluorescence from an appropriate amount of MCVs. We have expanded the quantification to two additional time points, at 3 h and 12 h after the start of the recording. The figure was amended to reflect the new results that conserved the trend observed at 6h in the other time points, which is no major difference in LC3 and LV intensity that was observed between the treated and untreated cells.

Finally, the title of Fig. 4 is "Autophagosomes are not bactericidal and IFN-γ treatment has no effect on LC3 recruitment frequency or phagosome maturation".

There is to the reviewer knowledge no evidence that autophagosomes are bactericidal, and since only LysoTracker blue staining was performed, it seems a bit overstated to conclude that there is no maturation.

The title of figure 4 was amended to address this comment and be more descriptive of the data, and to match better the revised conclusions.

As suggested previously, an additional dashed or coloured Y-line could be added in Fig5B-D and Y and X in FigE to indicate positivity. Please indicate here as well, how positivity was defined. If some rare events would appear with both LC3 and LysoView, as suggested by the positions of some individual dots in Fig 5E left panel, it would be interesting to select some of these peculiar events, and display some of their features by live-cell imaging in a main or supplementary figure, with access to the movies. These rare events, might by underrepresented but could be of broad interest for the community and therefore I would encourage the authors to display more about it.

For figure 5, the positivity sometimes used in the text in this context was defined by the dynamics of the different markers shown in figure S5. Indeed, the raw MFI values were normalized between the maximum and the minimum value recorded for each individual MCV, which makes the fluctuation of MFI comparable across the MCVs. Thus, it is possible to observe that for most of the tracked MCVs, the LV signal will drop to a level between 0 and 20% from the maximum intensity which we consider a loss of signal (or positivity). To avoid confusion, the results description was amended to mention signal intensity fluctuations rather than binary classification: line 269: "The resulting dot plot and density map clearly show two populations of stained MCVs, the ones exhibiting signal for LC3 (from 40-100% of normalized intensity), and ones showing a signal for LV (from 40-100% of normalized intensity) with very transient overlap between the 2 markers (Figure 5E)."

Conceptually, the rarity and the transient property of the concomitant staining in our experiments strongly suggest that the autophagy response is ineffective in its acidification process, potentially due to damage of this compartment as previously observed (Bernard et al 2017) and observed too in our experiments (figure 6a). We believe that from this observation, the future main interest would be to understand how these TVS are formed and why they don't acidify.

In Fig6, the authors investigate whether LC3 positive structures might be as well positive for galectin3, ALIX or OSBP, three markers widely used for more damage and repair processes. It is not clear why the authors did not perform this assay overtime, knowing that LC3 positivity fluctuates overtime, and starts very early during the infection kinetics. Can the authors comments about this?

Interestingly, at this specific time point, a larger number of LC3 (+) structures were reported to be associated with Galectin3 or OSBP (~2-5 fold), whereas very few LC3-ALIX double positive events were observed.

Have the authors considered looking at the ESCRT-III effector CHMP4 as well or other repair markers? Knowing that the temporal recruitment/dynamics of these markers can be interconnected and eventually precede one to each other, I would recommend the author to look into earlier additional timepoints (2hours pi, 6hours pi and 24hours pi) and eventually try to perform some additional live-cell imaging in transfected THP1-LC3 GFP cells to further support their conclusion. In the recent years, the concept of minor lysosomal lesion vs rupture has appeared. In that context, several protein actors are involved, and here a more thorough investigation of which one is involved, and when; seems to be crucial to really delve into LC3 positivity and its function in concert with other actors during Mtb infection/pathogenesis.

Based on this comment, the immunofluorescence approach was repeated and extended to 1 h, 6 h, and 24 h timepoints as suggested. The antibodies were validated by checking their ability to colocalize with LC3-GFP signal upon LLOME treatment that induces lysosomal membrane damage. While Galectin-3 and OSBP showed the expected punctate staining after treatment (Figure S8), the ALIX antibody did not (data not shown), thus its associated results were removed from the manuscript. We tested instead the CHMP4B antibody described here (DOI: 10.1128/mbio.01765-18) and we observed the expected phenotype upon LLOME treatment. The result of the treatment was added as a supplementary figure. The antibodies were then used on Mtb infected cells and the results for the different time points are now presented in the updated Figure 6. These results confirmed that OSBP but not CHMP4B was associated in majority with LC3+ MCVs. CHMP4B was still seen to be recruited but more on MCV without LC3 recruitment.

Finally, if the WB presented in FigS6 are convincing, a functional validation of the knock down would have been important to really validate that the pathway is not functional. This could be autophagic flux experiments, or immunofluorescence upon specific stimulation to further confirm that the Knockdown are effective before conducting experiments on Mtb. This validation step is lacking, and its difficult to trust the findings/conclusion of the Mtb experiments without the appropriate validation. In addition, pushing the replication towards 48h-72h would be interesting as monitoring growth/infectivity and cell-death over 24hours, is very limiting. The authors could really benefit from performing longer experiments and exploring additional timepoint to observe some events later in the kinetics.

For the Knock-Down approach, the ATG7 silencing displayed a decreased induction of LC3 recruitment on the MCV that we considered reasonable evidence for a functional knock-down.

Longer experiments were attempted but the cell death level after 24 h reaches level close to a 100% cell death observed. This limitation is not unexpected from THP-1 cells that can be more sensitive to Mtb infection than primary human macrophages. With our recently optimized model of primary human macrophage transiently transfected to express LC3, the study of the impact of KD of autophagy effectors or membrane repair effectors will be explored on long term time-lapse microscopy approaches.

Overall, it is an interesting story that requires some additional control experiments, some clarifications regarding the methodologies and approaches used, and I would personally recommend the authors to focus more onto the mechanistical aspects (from the host or the pathogen) of such recruitment and repair rather than just some observations/descriptions, as this would greatly benefit the findings for our community.

We thank the reviewer for this positive evaluation of our work and for the constructive criticisms.

Reviewer #2: 1. The authors should consider that change in phagosome acidification might occur for reasons other than phagosomal damage (e.g. H+ can be consumed in luminal reactions, altered by counter-ion flux, leakage, neutralized by the bacilli, etc.…see PMID: 33505976). If they use a bacterial mutant that is defective in Esx-1 function, what are the dynamics of acidification?

While Mtb is described as interfering with phagosome maturation, the few studies monitoring the phagosomal pH of MCVs described a pH stabilizing at around 6.5, which is the optimal one for Mtb growth but would still be detected as acidic by lysotracker-like probes such as Lysoview. An abrupt loss as measured within one or 2 frames followed by autophagy is likely following the model of phagosomal membrane damage followed by xenophagy. And for the results in figure 5, none of the MCVs observed were showing a loss of LV without LC3 recruitment, reinforcing the idea that this is membrane damage. Immunofluorescence staining confirmed the presence of GAL3, that was observed before (ref Bernard et al 2017). The leakage mentioned by the reviewer can be a consequence of membrane damage and this was mentioned in the discussion (line 408-421).

Following this comment, we also performed time-lapse monitoring of Wt Mtb and the *EsxA*-KO mutant (Figure S7D-G). We observed that the acidification dynamics between the strains only different in the first 2 h before stabilizing at the same level.

Reviewer #3: The authors draw conclusions based on experiments performed in only THP-1s, a macrophage-like immortalized cell line that doesn't always faithfully replicate macrophage responses to Mtb. To firmly establish the validity of these results, the authors should perform key live cell imaging experiments in additional macrophage-like cell lines or primary macrophages. While use of human macrophages is obviously preferable, much work on macrophage responses to Mtb has been performed using mouse macrophages, so utilizing mouse macrophages in order to assess primary cells, use fluorescent reporters, or establish that findings are generalizable would be reasonable.

The reviewer makes a good point. Following this comment, we replicated the analysis of GFP-LC3 / lysoview dynamics in primary human monocytes-derived macrophages (hMDMs). Overall similar dynamics were observed and are now reported in the new Figure S7.

I'm not convinced the authors establish through their data that "there is no major role of autophagy in cell autonomous defense against Mtb" since they do not directly examine Mtb viability or examine time points beyond 20 hr of imaging when Mtb killing might occur. I think this conclusion of the paper needs to be softened to better align with the data presented, or additional data supporting this claim should be included.

We agree with the reviewer's comment and the conclusion concerning the bactericidal properties of the LC3-TVS in the main text was softened.

The authors perform a careful analysis of the timing of initial recruitment events, but based on the Methods, it seems infections are not synced via spinfection. As a result, it's hard to establish the time of phagocytosis if bacteria could be phagocytosed at various times post-infection as they settle by gravity onto the macrophages. The authors should be mindful of this caveat when describing the timing of events in the Results.

We are aware of this potential caveat. However, for all of the time-lapse studies we can follow MCV from the time of phagocytosis onward and hence we can synchronize events by using the time of phagocytosis as the starting point.

Figure 1 was cut off in the submission PDF, so the data associated with it could not be evaluated.

Part III – Minor Issues: Editorial and Data Presentation Modifications

Please use this section for editorial suggestions as well as relatively minor modifications of existing data that would enhance clarity.

Reviewer #1: Minor Points

- Please add the appropriate reference for the Gobal TB report from the WHO.

- Please include the exact procedure that was carried out for nucleofection within the Material and Methods section instead of redirecting towards “The procedure for the nucleofection of siRNA was done following the neon transfection manufacturer’s instructions”. Also include the reference of the siRNA that have been used for OSBP and ATG7 since their respective references are missing.

We added the requested information.

- In panel Fig4. D-E-F it is not clear what the authors did not report the standard deviation as before?

- Same comment regarding the Fig S7?

For this data, the overlay of the 3-4 data points with errors bars tend to overcrowd the graph while the distribution of all the data points is visible. This type of representation is not clear for the histogram representation combining the multiple markers. This choice was made when multiple curves were overlayed.

- The use of one unique font used for the main figures throughout the manuscript is recommended as for some figures multiples fonts are mixed.

Thanks for pointing out these inconsistencies which we fixed for the revised manuscript.

Reviewer #2: 1. I don’t think the authors can make the conclusion, as stated, in their abstract, “Our data suggest that there is no major role of autophagy in cell autonomous defense against Mtb nor membrane repair.” They looked at bacterial burden only up to 20 hours, and the pH change is an indirect measure that they are interpreting as reflecting repair.

This conclusion was softened accordingly in the manuscript.

2. The conclusion that ALIX is not recruited to Mtb phagosomes requires that the ALIX antibody works in their conditions—i.e. it would be important to see a positive control that ALIX is recruited in their cell type, with their fixation method, etc. after lysosomal damage. Also, the findings on ALIX and OSBP are just from a late time point, so may not speak to the earlier events (e.g. 1-6 hours in Fig. 2). Finally, their discussion blurs the distinction between minor and more substantial damage which might require different repair mechanisms.

As stated previously for reviewer 1: the immunofluorescence approach was repeated and extended to 1 h, 6 h, and 24 h timepoints as suggested. Antibodies were validated by checking for their ability to colocalize with LC3-GFP signal upon LLOME treatment to induce lysosomal membrane damage. While Galectin-3 and OBSP showed the expected punctate staining after treatment (Figure S8), the ALIX antibody did not

(data not shown) thus its associated results were removed from the manuscript. We tested instead the CHMP4B antibody described here (DOI: 10.1128/mbio.01765-18) and we observed the expected phenotype upon LLOME treatment. The result of the treatment was added as a supplementary figure. The antibodies were then used on Mtb infected cells and the results for the different time points are now presented in the updated Figure 6. These results confirmed that OSBP but not CHMP4B was associated in majority with LC3+ MCVs. CHMP4B was still seen to be recruited but more on MCV without LC3 recruitment.

3. Since LC3-positivity always follows acidification, consider the role of the V-ATPase in non-canonical autophagy processes in which raised pH drives V-ATPase assembly and ATG16L1 recruitment (PMID: 36288298). More mechanistic insight could be gained around these observations, for example, by blocking acidification.

This possibility of LC3 conjugation of single membrane or atg8ylation was considered and discussed in the paragraph line 480. The fact that LC3 recruitment on mycobacteria is characterized by a double/multi-membrane structure argues against a triggering of LC3 recruitment by the V-ATPAse-ATG16L1 pathway as this atg8ylation is described on the single membrane of the phagosome. A potential contribution of this pathway to our observed phenomenon will be the subject of future research.

4. How do the authors envision that LC3 is rapidly lost from phagosomes, and how would that differ in egress vs exclusion. When the authors say, "quickly diffused away," could they be more specific? How long, on average, does that take? Is it different for egress vs exclusion? Do the dynamics provide any insight into how this might happen? Consider PMID: 33686057

For the egress, we envision that the bacteria physically dissociate from the autophagosome as described Bernard et al, 2020, where the bacteria end up residing in the cytosol. For the exclusion, the main hypothesis is that the recruitment of LC3 is "resolved" due to membrane repair. Considering the provided reference, we believe that non-canonical autophagy and ROS are not involved in the observed processes. The presence of membrane damages labeled by GAL3 support for a LC3 recruitment mediated by xenophagy. This is discussed in our main text as well (see line 433-452).

5. There are some inconsistencies with the literature – e.g. on the role of Atg7 in repair, that aren't explained or fully investigated (e.g. corroboration with additional methods of blocking autophagy, etc.). The impact of IFN-□ is another example, which might be related to cell type and the substantial cell death that

The role of ATG7 in membrane repair is, to our knowledge, not established or potentially linked to non-canonical autophagy that we believe to not be involved in the observed LC3 recruitment. The discussion focused instead on the potential role of canonical autophagy in membrane repair, and largely unknown during bacterial infections. The discussion part also proposed explanation on the effect observed with the IFN-γ treatment (line 433).

6. . In the images (Fig. 6A), OSBP signal appears to be within the LC3+ signal. Is this expected?

Some higher resolution imaging (airyscan) was performed and seems to show that in some instances, Gal3 and OSBP is indeed seen tight around the bacteria and extended within the LC3+ (not shown). This appears to be due to potential membrane damages extended to the LC3-TVS as observed with the galectin 3 in our study and 8 in Bernard et al., 2020.

7. The discussion is a bit long and difficult to read.

We reorganized the discussion and hope that it facilitates the reading experience.

Reviewer #3: Several sections of the Results paper were difficult to read and understand due to typos or overly clunky language. The authors should consider editing the text with an eye toward clarity so that the reader can more easily understand the experiments, the rationale, the analysis, etc.

We hope that the revised version is free of typos and less clunky.

The results from IFN-g treatment are surprising, and in examining the microscopy images, it seems the IFN-g-treated cells have far more bacteria in the fields shown. Is the increased cell death simply due to differences in MOI?

The bacterial burden between the untreated and treated cells revealed no difference as shown in the new Figure 4E (previously 4F).

It's not clear why ATG7 was chosen for KD to test if autophagy is required for membrane repair. Are other ATGs also detrimental to membrane repair? Is this detrimental effect of autophagy specific to Mtb infection (and bacterial virulence strategies) or does it also occur with sterile membrane damage?

ATG7 was chosen for its central role in autophagy, including its involvement in both canonical and non-canonical autophagy pathways. The role of ATG proteins, and autophagy more broadly, in membrane repair is still not fully understood. For example, ER-dependent membrane repair, which operates through lipid transfer to the damaged compartment, requires ATG2A independently of canonical autophagy pathways (DOI: 10.1038/s41586-022-05164-4). In cases of sterile membrane damage, ATG8ylation on single membranes appears to contribute to repair, though macroautophagy is more typically associated with the clearance of damaged compartments rather than direct repair. Our work here attempts to focus on the observed role of autophagy through the dynamics of LC3 recruitment, but future investigations will focus on the deciphering of the membrane repair mechanism of the phagosome.

The authors refer to their cells as expressing LC3-GFP, which implies the GFP is fused to the C-terminus of LC3. If this is the case, it interferes with LC3 function. As such, most reporters are GFP-LC3. The authors should clarify in the text and the Materials & Methods.

The cell line and its construct is now detailed in the method section line 568 : "THP-1 cell lines expressing GFP-N-terminal LC3B fusion protein used in a previous study,[50] were provided by the lab of Dr. John Kehrl (NIH) and prepared using a plasmid (pMXs-IP-GFP-LC3) provided by the lab of Dr. Noboru Mizushima (University of Tokyo). "

In Fig 4B and similar figures, the colors are too similar to one another, and the ordering of colors in the bar graph is not consistent with their or with their order in the legend, making it difficult to read and interpret.

The colors were adjusted to increase clarity on the histogram.

The authors use the phrase “minimal to none” when the data indicates that “minimal” is the accurate description; they should not say “none” if in fact there is some.

This inaccuracy was addressed in the revised manuscript.

The abstract conveys mixed messages about the involvement of and requirement for autophagy in membrane repair; I think this could be edited to be clearer and more consistent with the data presented.

The abstract was modified to clarify the lack of requirement of autophagy in membrane repair.

It may be beyond the scope of this manuscript, but it might be worth mentioning in the Discussion that LC3 is the terminal marker of autophagy; it would be interesting to examine the dynamics of intermediate markers of autophagy, like p62/NPD52, or sensors of membrane damage like galectin-3.

This is a relevant suggestion, but we agree that this is beyond the scope of the current study and the discussion.
